# Discovery and molecular characterization of a potent thiazolyl-pyrazole hybrid targeting EGFR for breast cancer therapy

**DOI:** 10.1038/s41598-025-07261-6

**Published:** 2025-07-03

**Authors:** Samar E. Mahmoud, Ahmed A. Fadda, Ehab Abdel-Latif, Mohamed R. Elmorsy

**Affiliations:** Department of Chemistry, Faculty of Science, Mansoura University, Mansoura, 35516 Egypt

**Keywords:** Thiazolyl-pyrazole analogues, Breast cancer, ADME, PPI network, Molecular Docking, EGFR, Molecular dynamics, Biochemistry, Structural biology

## Abstract

Herein, a novel compound 2-(3-methyl-5-oxo-1-phenyl-1,5-dihydro-4*H*-pyrazolylidene)-3-phenylthiazolidin-5-one )**2**(was synthesized and reacted with different aromatic aldehydes **3a-f** *via* Knoevenagel condensation reaction to give the corresponding 4-arylidene-2-(5-oxo-1,5-dihydro-4*H*-pyrazol-4-ylidene)thiazolidin-5-one hybrids **4a-f**. The chemical structures were described by spectroscopic tools, IR, ^1^H NMR, ^13^C NMR, and MS. Their frontier molecular orbitals configuration and electron distribution were estimated to utilize DFT. The cytotoxicity of thiazolyl-pyrazole analogues **2** and **4a-f** demonstrated in *vitro* antitumor activity toward breast cells; MCF-7 and MDA-MB231. Among the prepared analogues, the thiazolyl-pyrazole **2** revealed potent inhibitory toward the two cancer cells, particularly MDA-MB231 (IC_50_ = 22.84 µM). SwissADME studies showed the pharmacokinetic parameters, drug-like qualities, and bioavailability of these derivatives, revealing their potential in anticancer applications. Additionally, disease and drug target predictions and construction of the protein-protein interaction (**PPI**) network identified **PPARG**, **EGFR**, and **PPARA** as major targets. Moreover, other studies were carried out on the most potent conjugate **2** to evaluate the potential interactions against **PPARG**, **EGFR**, and **PPARA** proteins for molecular docking and against **EGFR** only for molecular dynamic simulation. The mechanism of the most effective analogue **2** was proven experimentally by inhibiting wound healing and EGFR expression in MDA-MB231 culture media. The findings provide more credence to compound **2**’s potential in current medication development initiatives.

**Supplementary Information:**

The online version contains supplementary material available at 10.1038/s41598-025-07261-6.

## Introduction

Breast cancer appears when breast cells begin to grow out of control. In spite of, ongoing amelioration in medical, it remains the most widespread cancer among females^1^. It remains an enormous adversary in the landscape of global health challenges, with its complicated pathogenesis posing significant obstacles^2,3^. The prognosis of many breast cancers is based on an X-ray scan by looking closely at breast tissues. At diagnosis, rarely the first sign of this type is changes in the skin or nipple of the breast only^4^. But breast cancer is also found in the axillary lymph nodes under the arms and other areas in the body. The stage of this illness is assigned based on where the cancer is found. The most dangerous stage is IV; it is stage that breast cancer is spread to different places in the body^5^. Breast cancer is split into three kinds based on the presence or obscurity of different proteins in breast cells^6^. Globally, there were about 2.3 million new cases of breast cancer in 2020, and about 685,000 people died^7^. By 2040, it has been expected that there will be more than 3 million new infections each year with 1 million annual deaths due to population growth rate and ageing^8^.

**Fig. 1:**
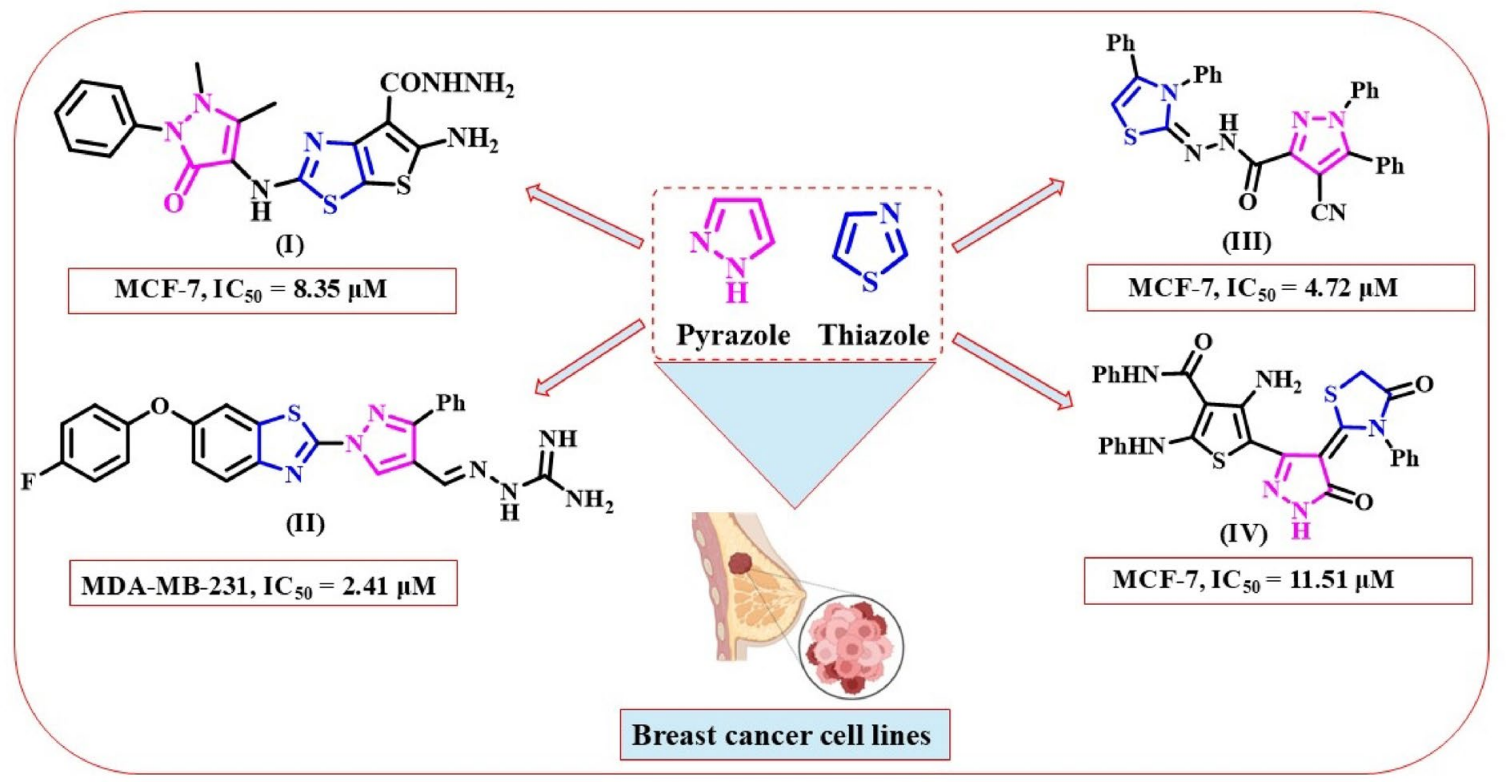
Reported examples of some thiazole integrated with pyrazole as breast cancer agents.

Heterocyclic compounds, especially those that contain nitrogen and sulfur atoms, are considered one of the most significant categories of organic compounds used in various biological fields owing to their action on a range of diseases^9^. There are numerous heterocyclic moieties such as pyrazole, pyrrole, thiazole, and thiazine which present the backbone of medicines^10^. For example, pyrazole ring containing two nitrogen atoms has attracted considerable attention recently because of its less toxic effects and several pharmacological features^11,12^. Additionally, thiazole moiety is a multilateral lead molecule in pharmaceutical growth and is clinically used in various diseases^13^. A literature search detected that fusing pyrazole with five-membered ring thiazole is key to the synthesis of a new series of organic compounds that are effective against biological activities, particularly breast anti-cancer^14,15^. On the other hand, different aromatic aldehydes (triphenylamine, 3,4,5-trimethoxybenzene, and *N*,* N*-dimethylaniline) and heteroaromatic aldehydes (phenothiazine and indole) have versatile therapeutic applications^16–20^. For instance, in 2022, Othman et al.. designed different series of thiazolyl-pyrazole derivatives. They were further investigated for MCF-7 and HepG-2 cells, where the derivative of the compound (**I**) displayed IC_50_ values of 8.35 µM and 7.88 µM, respectively^21^. Furthermore, in 2019, Liu et al.. prepared novel pyrazole ring with benzothiazole derivatives. Type (**II**) was also tested against different breast cell lines, MCF-7 and MDA-MB-231 and showed good values of IC_50_ = 2.23 µM and 2.41 µM, respectively^22^. Moreover, in 2010, Farag et al.. synthesized a series of 1,5-diphenylpyrazole derivatives. Among the examined derivatives toward breast cancer, derivative (**III**) exhibited IC_50_ = 4.72 µM with MCF-7 and IC_50_ = 7.36 µM with MDA-MB-231, respectively^23^. In 2021, Masaret et al.. synthesized thiazolylidenepyrazolyl thiophene systems and determined cytotoxicity toward the MCF-7 and HepG2 cell lines where analogue (**IV**) displayed IC_50_ values (11.51 µM and 27.62 µM), respectively^24^. Structures of reported compounds are demonstrated in Fig. 1.

The vast spectra of the biological profile of the thiazolyl-pyrazole system attracted the attention of our research group to accommodate thiazole and pyrazole moieties in a single molecular framework to produce some new heterocyclic compounds. The anticancer efficiency of these new hybrids was investigated using the MTT assay across MCF-7 and MDA-MB-231 cell lines. The prepared compounds were also subjected to modeling, molecular docking, and molecular dynamics. Furthermore, Swiss ADME estimated drug-likeness of all derivatives.

## Experimental section

### Materials and apparatus

All melting points (m.p.) were obtained using a Gallenkamp device and were uncorrected. The IR spectra were run on Bruker Alpha II FTIR spectrometer (Germany). The spectra of NMR were recorded on JEOL ECA II and Bruker Avance III at 500 MHz and 400 MHz for^1^H NMR and 125 MHz and 100 MHz for^13^C NMR, respectively, using (TMS) as an internal standard and DMSO-*d*_6_ as a solvent. The mass spectra (EI) were performed through Thermo Fisher Scientific GC/MS model DSQ II with 70 eV. The Perkin-Elmer 2400 analyzer was used to gather the elemental analyses (C, H, and N).

### Synthesis of 2-(3-methyl-5-oxo-1-phenyl-1,5-dihydro-4*H*-pyrazol-4-ylidene)-3-phenylthiazolidin-5-one (2)

A mixture of 5-methyl-2-phenyl-2,4-dihydro-3*H*-pyrazol-3-one (**1**) (1.74 g, 10 mmol), phenyl isothiocyanate (1.20 mL, 10 mmol) and potassium hydroxide (0.56 g, 10 mmol) was stirred in 20 mL DMF for 6 h. Then chloroacetyl chloride (1.19 mL, 15 mmol) was added with continuous stirring for additional 6 h. The mixture was then diluted with 50 mL of cold water. The formed solid with was filtered, washed with H_2_O, and recrystallized from acetic acid to yield the conforming pyrazolyl-thiazolidin-5-one **2**.

Greenish yellow solid, yield = 85%; m.p. = 262–264 °C. IR (*ῡ*): 2974 and 2931 (C-H, sp^3^), 1735 1650 (C = O), 1591 (C = C) cm^−1^. ^1^H NMR (*δ*): 0.96 (s, 3 H, CH_3_), 4.17 (s, 2 H, CH_2_), 7.13 (t, *J* = 8.0 Hz, 1 H, Ar-H), 7.37 (t, *J* = 8.0 Hz, 2 H, Ar-H), 7.48–7.49 (m, 2 H, Ar-H), 7.54–7.59 (m, 3 H, Ar-H), 7.86 (d, *J* = 7.5 Hz, 2 H, Ar-H) ppm. ^13^C NMR (*δ*): 16.41, 31.77, 102.15, 117.90 (2 C), 118.43 (2 C), 124.26, 128.87 (2 C), 129.81 (2 C), 137.30, 138.38, 143.40, 165.67, 168.22, 174.25 ppm. Analysis for C_19_H_15_N_3_O_2_S (349.09): Calculated: C, 65.31; H, 4.33; N, 12.03%. Found: C, 65.50; H, 4.28; N, 12.13%.

### Synthesis of 4-arylidene-2-(5-oxo-1,5-dihydro-4 H-pyrazol-4-ylidene)thiazolidin-5-one derivatives 4a-4f

A solution of 2-(3-methyl-5-oxo-1-phenyl-1,5-dihydro-4*H*-pyrazol-4-ylidene)-3-phenylthiazolidin-5-one (**2**) (0.69 g, 2 mmol) and fused sodium acetate (0.32 g, 4 mmol) in glacial acetic acid (25 mL) was mixed with 2 mmol of each diverse aromatic aldehyde **3a**-**f**(namely; 4-(diphenylamino)benzaldehyde (0.54 g), pyrene-2-carbaldehyde (0.46 g), 10-ethyl-10*H*-phenothiazine-3-carbaldehyde (0.51 g), 1-hexyl-1*H*-indole-3-carbaldehyde (0.45 g), 3,4,5-trimethoxybenzaldehyde (0.39 g), and 4-(dimethylamino)benzaldehyde (0.29 g)), respectively. The reaction mixture was heated for half an hour at 125 °C. The colored powder that formed on hot was collected, filtered, dried, and washed with boiling ethanol to offer purposed compounds **4a-4f**.

### 4-(4-(Diphenylamino)benzylidene)-2-(3-methyl-5-oxo-1-phenyl-1,5-dihydro-4*H*-pyrazol-4-ylidene)-3-phenylthiazolidin-5-one (4a)

Dark red solid, yield = 62%; m.p. > 310 °C. IR (*ῡ*): 2919 and 2842 (C-H, sp^3^), 1707 and 1650 (C = O), 1570 (C = C) cm^−1^^1^. H NMR (*δ*): 2.10 (s, 3 H, CH_3_), 7.03 (d, *J* = 8.8 Hz, 1 H, Ar-H), 7.17 (t, *J* = 7.2 Hz, 2 H, Ar-H), 7.22–7.26 (m, 5 H, Ar-H), 7.39–7.47 (m, 7 H, Ar-H), 7.64 (m, 5 H, Ar-H), 7.70 (d, *J* = 8.8 Hz, 2 H, Ar-H), 7.87 (s, 1 H, CH = C), 7.90 (d, *J* = 8.0 Hz, 2 H, Ar-H) ppm. ^13^C NMR (*δ*): 14.54, 104.23, 118.90 (2 C), 122.63, 122.99 (2 C), 124.10 (2 C), 125.48 (2 C), 126.00 (4 C), 126.98 (2 C), 128.10 (2 C), 128.58, 128.76 (4 C), 129.72 (2 C), 138.30, 139.76, 140.91, 141.56, 144.50, 144.97 (2 C), 145.42, 146.73, 152.00, 165.63, 168.17 ppm. Mass analysis (m/z, %): 604 (M^+^, 10.16), 573 (11.43), 440 (13.79), 411 (12.65), 391 (24.04), 371 (29.97), 267 (31.19), 227 (33.91), 117 (26.77), 116 (50.17), 84 (30.71), 83 (100.00), 81 (50.86). Analysis for C_38_H_28_N_4_O_2_S (604.19): Calculated: C, 75.47; H, 4.67; N, 9.26%. Found: C, 75.71; H, 4.59; N, 9.14%.

### 4-((10-Ethyl-10*H*-phenothiazin-3-yl)methylene)-2-(3-methyl-5-oxo-1-phenyl-1,5-dihydro-4*H*-pyrazol-4-ylidene)-3-phenylthiazolidin-5-one (4b)

Dark red solid, yield = 58%; m.p. = > 300 °C. IR (*ῡ*): 2922 and 2855 (C-H, sp^3^), 1708, 1662 (C = O), 1586 (C = C) cm^−1^. ^1^H NMR (*δ*): 1.33 (t, *J* = 7.0 Hz, 3 H, CH_3_), 1.87 (s, 3 H, CH_3_), 3.99 (q, *J* = 7.0 Hz, 2 H, CH_2_), 6.99 (t, *J* = 7.0 Hz, 1 H, Ar-H), 7.08 (d, *J* = 8.0 Hz, 1 H, Ar-H), 7.16 (t, *J* = 7.5 Hz, 2 H, Ar-H), 7.21 (d, *J* = 8.0 Hz, 1 H, Ar-H), 7.24 (d, *J* = 9.0 Hz, 1 H, Ar-H), 7.40 (t, *J* = 8.0 Hz, 2 H, Ar-H), 7.52 (d, *J* = 2.5 Hz, 1 H, Ar-H), 7.59–7.61 (m, 5 H, Ar-H), 7.64–7.66 (dd, *J* = 2.5, 4.0 Hz, 1 H, Ar-H), 7.83 (s, 1 H, CH = C), 7.88 (d, *J* = 8.0 Hz, 2 H, Ar-H) ppm. ^13^C NMR (*δ*): 12.70, 13.32, 42.46, 104.10, 114.12, 115.33, 119.17 (2 C), 120.00, 121.91, 122.10 (2 C), 123.04, 123.53, 124.68, 127.36, 127.45, 127.53, 128.67 (2 C), 129.99 (2 C), 132.56, 134.73, 138.07, 141.52, 142.20, 143.87, 145.38, 147.12, 148.87, 150.84, 162.26, 166.32 ppm. Mass analysis (m/z, %): 586 (M^+^, 17.69), 561 (30.99), 542 (26.67), 520 (31.33), 502 (45.05), 472 (23.06), 468 (43.32), 408 (29.53), 372 (34.55), 356 (44.50), 344 (62.64), 259 (28.94), 196 (27.52), 140 (42.39), 104 (29.22), 52 (100.00). Analysis for C_34_H_26_N_4_O_2_S_2_ (586.15): Calculated: C, 69.60; H, 4.47; N, 9.55%. Found: C, 69.38; H, 4.40; N, 9.42%.

### 2-(3-Methyl-5-oxo-1-phenyl-1,5-dihydro-4*H*-pyrazol-4-ylidene)-3-phenyl-4-(pyren-2-ylmethylene)thiazolidin-5-one (4c)

Pale red solid, yield = 51%; m.p. = > 330 °C. IR (*ῡ*): 2923 and 2865 (C-H, sp^3^), 1706 and 1660 (C = O), 1588 (C = C) cm^−1^. ^1^H NMR (*δ*): 1.89 (s, 3 H, CH_3_), 7.13–7.15 (m, 2 H, Ar-H), 7.36–7.40 (m, 3 H, Ar-H), 7.48 (d, *J* = 7.0 Hz, 1 H, Ar-H), 7.56 (d, *J* = 7.0 Hz, 2 H, Ar-H), 7.65 (d, *J* = 8.0 Hz, 1 H, Ar-H), 7.71 (d, *J* = 7.5 Hz, 1 H, Ar-H), 7.86 (d, *J* = 7.0 Hz, 3 H, Ar-H), 8.16–8.20 (m, 1 H, Ar-H), 8.30 (d, *J* = 8.5 Hz, 1 H, Ar-H), 8.37 (d, *J* = 9.5 Hz, 1 H, Ar-H), 8.41–8.48 (m, 2 H, Ar-H), 8.55 (t, *J* = 9.0 Hz, 1 H, Ar-H), 8.92 (s, 1 H, CH = C) ppm. ^13^C NMR (*δ*): 14.65, 104.19, 119.30 (2 C), 122.42, 122.95, 123.61 (2 C), 124.69 (2 C), 125.84, 126.20, 127.07 (2 C), 127.46, 127.97 (2 C), 128.11, 128.91 (2 C), 129.52 (2 C), 129.90 (2 C), 131.34, 132.01, 135.61, 138.08 (2 C), 140.15, 144.59, 146.38, 149.31, 153.22, 166.59, 168.45 ppm. Mass analysis (m/z, %): 561 (M^+^, 90.74), 556 (14.37), 552 (13.80), 499 (18.94), 462 (24.62), 445 (26.35), 396 (12.22), 287 (26.29), 212 (27.12), 182 (43.33), 165 (100.00), 150 (49.44), 145 (37.12), 141 (54.74), 106 (48.02), 98 (49.34), 43 (89.75). Analysis for C_36_H_23_N_3_O_2_S (561.15): Calculated: C, 76.99; H, 4.13; N, 7.48%. Found: C, 76.81; H, 4.20; N, 7.58%.

### 4-((1-Hexyl-1*H*-indol-3-yl)methylene)-2-(3-methyl-5-oxo-1-phenyl-1,5-dihydro-4*H*-pyrazol-4-ylidene)-3-phenylthiazolidin-5-one (4d)

Orange solid, yield = 45%; m.p. = 202–204 °C. IR (*ῡ*): 2922 and 2856 (C-H, sp^3^), 1661 (C = O), 1587 (C = C) cm^−1^. ^1^H NMR (*δ*): 0.82 (t, *J* = 7.5 Hz, 3 H, CH_3_), 1.17–1.31 (m, 6 H, -CH_2_-CH_2_-CH_2_-), 1.82–1.88 (m, 2 H, CH_2_), 2.40 (s, 3 H, CH_3_), 4.38 (t, *J* = 7.5 Hz, 2 H, CH_2_), 7.04 (t, *J* = 8.0 Hz, 1 H, Ar-H), 7.15 (t, *J* = 7.0 Hz, 1 H, Ar-H), 7.34–7.38 (m, 5 H, Ar-H), 7.42 (t, *J* = 8.0 Hz, 3 H, Ar-H), 7.69 (d, *J* = 8.5 Hz, 1 H, Ar-H), 8.0 (d, *J* = 7.00 Hz, 3 H, Ar-H), 8.07 (s, 1 H, CH = C), 8.17 (d, *J* = 8.5 Hz, 1 H, Ar-H), 9.82 (s, 1 H, Ar-H) ppm. ^13^C NMR (*δ*): 13.49, 14.29, 22.45, 26.19, 29.71, 31.13, 47.37, 104.20, 110.90, 111.91, 112.07, 118.60 (2 C), 118.63 (2 C), 119.45 (2 C), 123.05 (2 C), 124.22, 124.55 (2 C), 129.26 (2 C), 136.95, 137.18, 139.29, 140.81, 146.18, 147.98, 151.54, 153.12, 163.30, 167.31 ppm. Mass analysis (m/z, %): 560 (M^+^, 9.71), 551 (23.25), 504 (26.27), 450 (32.12), 447 (34.56), 444 (38.62), 433 (53.13), 425 (43.63), 405 (41.65), 391 (61.98), 386 (100.00), 376 (30.79), 316 (60.99), 232 (44.40), 230 (45.02), 208 (44.06), 206 (35.85), 194 (39.18), 179 (26.67). Analysis for C_34_H_32_N_4_O_2_S (560.22): Calculated: C, 72.83; H, 5.75; N, 9.99%. Found: C, 72.58; H, 5.84; N, 9.87%.

### 2-(3-Methyl-5-oxo-1-phenyl-1,5-dihydro-4*H*-pyrazol-4-ylidene)-3-phenyl-4-(3,4,5-trimethoxybenzylidene)thiazolidin-5-one (4e)

Orange solid, yield = 48%; m.p. = 270–272 °C. IR (*ῡ*): 2925 and 2851 (C-H, sp^3^), 1720 and 1656 (C = O), 1576 (C = C) cm^−1^. ^1^H NMR (*δ*): 2.01 (s, 3 H, CH_3_), 3.77 (s, 3 H, OCH_3_), 3.88 (s, 6 H, 2 OCH_3_), 7.15 (s, 2 H, Ar-H), 7.40 (t, *J* = 8.0 Hz, 3 H, Ar-H), 7.60–7.62 (m, 5 H, Ar-H), 7.85 (d, *J* = 8.0 Hz, 2 H, Ar-H), 7.90 (s, 1 H, CH = C) ppm. ^13^C NMR (*δ*): 15.93, 56.21 (2 C), 60.16, 101.94, 108.72 (2 C), 118.10 (2 C), 119.46, 124.32, 127.43, 128.59 (2 C), 128.82 (2 C), 129.58 (2 C), 129.81, 135.55, 137.96, 140.34, 143.62, 148.46, 153.16 (2 C), 158.56, 165.18, 166.31 ppm. Mass analysis (m/z, %): 527 (M^+^, 12.90), 516 (34.17), 505 (37.47), 480 (46.23), 461 (75.96), 453 (64.57), 401 (51.32), 350 (56.88), 348 (54.67), 342 (43.34), 331 (100.00), 277 (50.85), 274 (68.04), 266 (77.37), 248 (59.17), 225 (53.40), 143 (82.85), 140 (46.35), 119 (57.15), 91 (73.04). Analysis for C_29_H_25_N_3_O_5_S (527.15): Calculated: C, 66.02; H, 4.78; N, 7.96%. Found: C, 66.17; H, 4.75; N, 7.88%.

### 4-(4-(Dimethylamino)benzylidene)-2-(3-methyl-5-oxo-1-phenyl-1,5-dihydro-4*H*-pyrazol-4-ylidene)-3-phenylthiazolidin-5-one (4f)

Red solid, yield = 55%; m.p. = 252–254 °C. IR (*ῡ*): 2915 and 2885 (C-H, sp^3^), 1664 and 1619 (C = O), 1548 (C = C) cm^−1^. ^1^H NMR (*δ*): 2.01 (s, 3 H, CH_3_), 3.06 (s, 6 H, -N(CH_3_)_2_), 6.83 (d, *J* = 8.5 Hz, 1 H, Ar-H), 6.91 (d, *J* = 8.5 Hz, 2 H, Ar-H), 7.15 (t, *J* = 7.5 Hz, 1 H, Ar-H), 7.39 (t, *J* = 9.0 Hz, 3 H, Ar-H), 7.47–7.53 (m, 3 H, Ar-H), 7.67 (d, *J* = 9.0 Hz, 2 H, Ar-H), 7.83 (s, 1 H, CH = C), 7.89 (d, *J* = 8.0 Hz, 2 H, Ar-H) ppm. ^13^C NMR (*δ*): 14.93, 41.64 (2 C), 104.14, 111.65 (2 C), 116.01, 119.80 (2 C), 121.45 (2 C), 122.23, 123.95, 125.99, 127.14 (2 C), 128.03 (2 C), 128.28 (2 C), 131.31, 142.40, 146.08, 147.28, 147.98, 150.20, 165.17, 167.84 ppm. Mass analysis (m/z, %): 480 (M^+^, 20.59), 442 (45.25), 404 (49.64), 383 (40.39), 380 (42.44), 352 (40.17), 329 (48.16), 294 (34.59), 245 (41.43), 225 (58.77), 181 (100.00), 175 (56.46), 158 (87.79), 155 (43.84), 149 (40.66), 100 (78.28), 77 (48.62). Analysis for C_28_H_24_N_4_O_2_S (480.16): Calculated: C, 69.98; H, 5.03; N, 11.66%. Found: C, 69.86; H, 5.05; N, 11.73%.

### Computational studies

#### DFT studies

The molecular geometry, electronic properties, and chemical reactivity descriptors of the synthesized derivatives were investigated in the gas phase based on DFT and by using B3LYP functional combined with basis sets 6-311G(d, p)^25^.

#### ADME profiling

The ADME, pharmacokinetic parameters, and drug-likeness of synthesized thiazolyl-pyrazole derivatives were evaluated using the free web tool, Swiss ADME (http://www.swissadme.ch)^26^. Furthermore, the potential oral bioavailability of these compounds was assessed using their radar charts. Also, the gastrointestinal permeability and the chemicals’ capacity to pass across the blood-brain barrier were predicted using the BOILED-Egg plots^27,28^.

#### Disease target prediction

The breast cancer targets were obtained from three databases: GeneCards (https://www.genecards.org/)^29^, CTD (http://ctdbase.org/)^30^, and DisGeNET (https://www.disgenet.org/)^31^. These databases are regularly updated online resources that provide extensive information on human genes and genetic disorders. The intersection between drug targets and disease targets was obtained utilizing the VENNY 2.1.0 online tool (https://bioinfogp.cnb.csic.es/tools/venny/)^32^ and a Venn diagram was generated.

#### Drug targets predictions

The molecular targets of the compound were predicted utilizing the web server PharmMapper (https://www.lilab-ecust.cn/pharmmapper/) which identifies the drug targets using a pharmacophore mapping approach. The predicted targets were downloaded, and only those with normalized Fit scores and Z-scores greater than 0.5 were chosen for further analysis.

#### Construction of PPI network

The protein-protein interaction (PPI) network for the common targets between the drug and the breast cancer was constructed using STRING database (https://string-db.org/)^33^. The species was set to “*Homo sapiens*” and all interacting pairs with a confidence score of 0.4 were imported into Cytoscape 3.10.2 software to demonstrate the gene interactions. CytoHubba, a tool within Cytoscape, identifies and analyses hub nodes within the network using various topological algorithms. Here, the Degree algorithm was used to identify the top ten hub genes.

### Anticancer evaluation

#### Cell culture

The human breast cancer cells MDA-MB-231 and MCF-7 were purchased from Naawh scientific co., originally obtained from the American Type Culture Collection (ATCC, USA). Cell lines were cultured in Dulbecco’s Modified Eagle medium (DMEM) supplemented with penicillin-streptomycin (100 U/mL) and 10% (v/v) fetal bovine serum FBS at 37 °C in a humidified atmosphere containing 5% carbon dioxide (CO_2_).

#### MTT assay (3-(4,5-dimethylthiazol-2-yl)-2,5-diphenyltetrazolium bromide)

MDA-MB-231 and MCF-7 cells were seeded in 96-well plates at a density of 5,000 cells per well and allowed to adhere for 24 h. The cells were then treated with varying concentrations of the tested compounds (100–6.25 µM), while control wells received 0.1% DMSO. After 48 h of incubation, the culture medium was taken away, and the cells were gently washed with phosphate buffered saline (PBS). Subsequently, 100 µL of fresh medium containing 0.5 mg/mL MTT (3-(4,5-dimethylthiazol-2-yl)-2,5-diphenyltetrazolium bromide) was added to each well, followed by incubation at 37 °C for 4 h. After incubation, the MTT solution was carefully discarded, and the wells were washed with PBS to eliminate any residual MTT or debris. The resulting crystals of formazan were dissolved by adding 100 µL of 100% DMSO to each well, and the plate was jolted for 30 min to ensure complete solubilization^34^. Finally, the absorbance was determined at 570 nm using an ELISA plate reader. Normalized cell viability was calculated by the following equation:

$$\:Cell\:viability\:\%=\frac{Abs\:of\:treated\:samples-Abs\:of\:blank\:control\:}{Abs\:of\:control\:samples-Abs\:of\:blank\:control\:} \times 100$$

#### Wound healing methods using MDA-MB-231 cancer cells

MDA-MB-231 cells were sowed in 6-well plates at a density of 2.5 × 10^5^ cells per well in complete media and allowed to adhere overnight under standard culture conditions. Once the monolayer reached 90–100% confluency, a uniform scratch was created in each well using a sterile 100 µL pipette tip to simulate a wound area. The wells were then gently washed with PBS to get rid of any cellular debris, and a fresh medium containing 0.5% serum was added to minimize proliferation effects. Cells were subsequently treated with compound **2** at 10 µM and 20 µM. Wound images were captured at 0 h (immediately after the scratch) and again at 48 h when the wound in the control group had nearly closed. The wound area was quantified at both time points using the ImageJ “Wound Healing Size Tool” plugin, and the percentage of wound closure at 48 h was computed relative to the initial wound area (0 h) for each treatment condition.

#### EGFR by ELISA

The EGFR (ERBB) Human ELISA Kit (Abcam, ab100505) was used to quantify EGFR levels in the cell culture supernatants of MDA-MB-231 cells treated with compound 2 (20 µM), erlotinib (positive control, 20 µM), and untreated control cells. MDA-MB-231 cells were sowed in six-well plates and incubated overnight under standard culture conditions. The next day, cells were treated with the respective compounds for 24 h, after which the culture media were collected and centrifuged at 3000 rpm for 10 min to remove debris.

The ELISA was performed following the manufacturer’s protocol. 100 µL of standards and samples were added to the pre-coated wells and incubated at room temperature for 2.5 h with gentle shaking. Wells were washed four times with buffer before adding 100 µL of biotinylated anti-human EGFR antibody, followed by incubation for 1 h. After washing, 100 µL of HRP-streptavidin solution was added and incubated for 45 min. The wells were again washed, and 100 µL of TMB substrate solution was added, incubating for half hour in the dark. The reaction was stopped by adding 50 µL of stop solution, and the absorbance was determined at 450 nm using a microplate reader. EGFR concentrations were computed based on a standard curve, and data were analyzed to compare the inhibitory effects of compound **2** and erlotinib.

### In silico studies

#### Molecular docking study

The 3-dimensional structures of known inhibitors of epidermal growth factor receptor (EGFR) namely Erlotinib^35^ Osimertinib^36,37^ and Neratinib^38,39^ were obtained from PubChem with CIDs 176,870, 71,496,458 and 9,915,743, respectively. The compounds’ structures were prepared for molecular docking and made flexible using Autodock Tools 1.5.7^40^.

The crystal structures of the human proteins peroxisome proliferator-activated receptor gamma (PPARG), epidermal growth factor receptor (EGFR), and peroxisome proliferator-activated receptor alpha (PPARA) were obtained from RCSB Protein Data Bank (https://www.rcsb.org/) with PDB IDs 9F7W, 1IVO and 6KB4, respectively. The preparation of the proteins’ structures in Autodock Tools involved removing water molecules and non-protein residues, adding polar hydrogens and assigning Kollman charges. The grid boxes coordinates were set based on the co-crystallized ligands. These ligands were extracted and saved as individual files to serve as standards for redocking experiments. This approach was implemented to validate the precision and reliability of the docking protocol.

Molecular docking of the flexible ligands and rigid proteins was performed using GNINA software v. 1.1^41^. Visualization of the docking outcomes was carried out using Discovery Studio 2021 and PyMOL v. 3.0.3.

#### Molecular dynamics simulation (MDS)

The topology parameters for the tested compound were generated using ACPYPE (AnteChamber PYthon Parser interfacE) version 2023.10.27 with the General Amber force field (GAFF). For the EGFR protein, the topology parameters were established using the AMBER99SB force field within the GROMACS 2024.2 package^42^. The protein-ligand complex was solvated in a triclinic box employing the simple point-charge (SPC) water model, and the system was neutralized by adding two Cl^-^ counterions. Energy minimization was carried out using the steepest descent algorithm for 50,000 steps, ensuring an Fmax below 100 kJ/mol. The minimized system underwent equilibration in two stages: first, under the NVT (canonical) ensemble using the V-rescale thermostat algorithm at 300 K for 200 ps, and then under the NPT (isothermal-isobaric) ensemble using the Parrinello-Rahman barostat algorithm for 500 ps. Eventually, MDS were conducted for 100 ns under the NPT ensemble with a time step of 2 fs. Long-range electrostatic interactions were computed by the Particle Mesh Ewald (PME) method, while hydrogen bond (H.B) lengths were constrained using the Linear calculated Solver (LINCS) algorithm.

Following the MDS, periodic boundary conditions were eliminated, and the trajectories were analyzed using GROMACS tools. These included **rms** for Root Mean Square Deviation (RMSD), **rmsf** for Root Mean Square Fluctuation (RMSF), **gyrate** for Radius of Gyration (RG), **sasa** for Solvent Accessible Surface Area (SASA), and **H-bond** to determine the number of hydrogen bonds.

## Results and discussion

### Chemistry

**Fig. 2:**
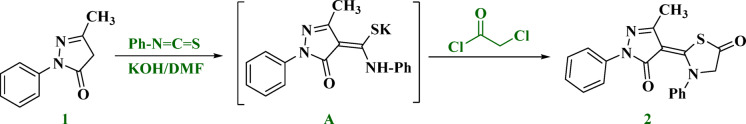
Synthetic route of 2-(3-methyl-5-oxo-1-phenyl-1,5-dihydro-4*H*-pyrazol-4-ylidene)-3-phenylthiazolidin-5-one (**2**).

**Fig. 3:**
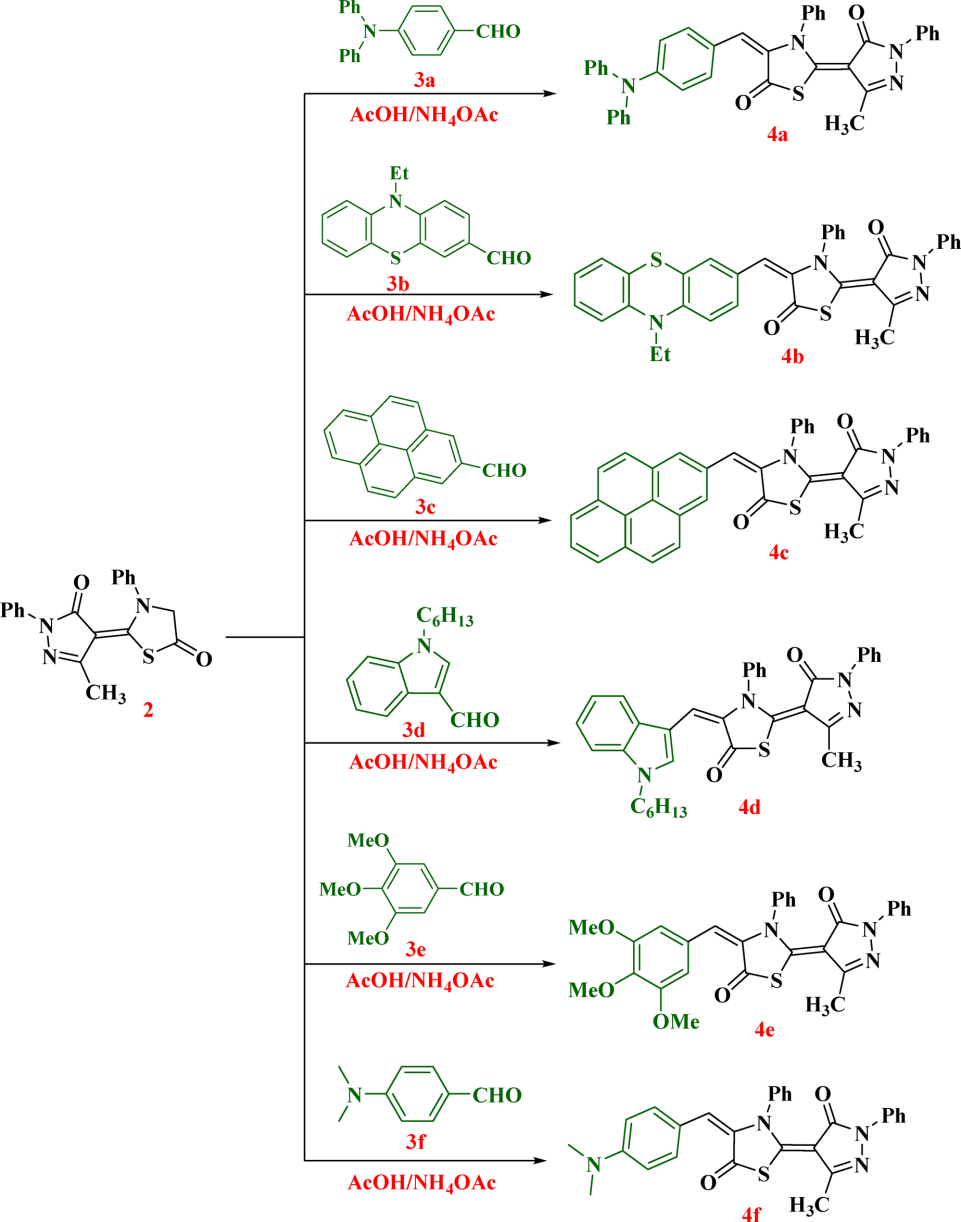
Synthesis of thiazolyl-pyrazole analogues **4a-4f**.

The synthetic routes of the synthesized thiazolyl-pyrazole analogues **4a–4f** are illustrated in Figs. 2 and 3. The preparation of the new thiazolyl-pyrazole hybrid **2** was achieved by stirring of 5-methyl-2-phenyl-2,4-dihydro-3*H*-pyrazol-3-one (**1**) with phenyl isothiocyanate in DMF in the presence of KOH to give non-isolable sulfide salt (**A**). This salt was cyclized in *situ* by chloroacetyl chloride to produce the corresponding thiazolyl-pyrazole hybrid compound **2**(Fig. 2).

Thereafter, compounds **4a-4f** with were obtained through the Knoevenagel condensation of 2-(3-methyl-5-oxo-1-phenyl-1,5-dihydro-4*H*-pyrazol-4-ylidene)-3-phenylthiazolidin-5-one (**2**) with different aromatic aldehydes and heteroaromatic aldehydes in the presence of ammonium acetate as catalyst and acetic acid, as summarized in Fig. 3. The molecular structures of thiazolyl-pyrazole analogues **4a-4f** were belayed with several spectral analyses. The IR spectrum of thiazolyl-pyrazole derivative containing triphenylamine unit **4a** showed characteristic absorption bands attributed to the carbonyl groups (C = O) at 1707 and 1650 cm^−1^. Moreover, stretching vibration bands of olefinic groups (C = C) exhibited at 1570 cm^−1^. The ^1^H NMR spectra of six derivatives **4a-4f** lacked any singlet signal at *δ* 4.17 ppm related to the methylene group of thiazolidin-5-one **2**, indicating condensation reaction and arylidene conjugates. Additionally, these compounds showed a distinctive singlet signal at the range of *δ* 7.83–8.92 ppm attributed to the olefinic group’s proton. The ^1^H NMR spectrum of analogue **4a** as an example revealed characteristic signals for the methyl group at *δ* 2.10 ppm and the aromatic protons at the range of *δ* 7.03–7.90 ppm. Furthermore, the molecular ion peak that occurred in the mass spectrum at m/z = 604 corresponded to the molecular formula C_38_H_28_N_4_O_2_S. The ^1^H NMR spectrum of compound **4b** exhibited distinguish signals for phenothiazine moiety, where the ethyl group (-CH_2_-CH_3_) appeared as triplet and quartet signals at *δ* 1.33 and 3.99 ppm, respectively. Further, the two distinctive protons of the PTZ unit (H2, H4) were observed as a doublet signal and a doublet of doublet signal at *δ* 7.21 ppm and 7.64–7.66 ppm, respectively. Also, ^13^C NMR proved the formed structure, where characteristic signals of methyl (CH_3_), ethyl (-CH_2_-CH_3_), and carbonyl (C = O) groups were resonated at *δ* 12.70, 13.32, 42.46, 162.26, and 166.32 ppm, respectively. The IR spectrum of derivative based on pyrene unit **4c** displayed vibration peaks of (C = O) groups at 1706 and 1660 cm^−1^. Furthermore, H NMR of hybrid **4f** gave a singlet signal at *δ* 3.06 ppm assigned to six equivalent protons of symmetrical methyl groups.

### DFT calculations

**Fig. 4:**
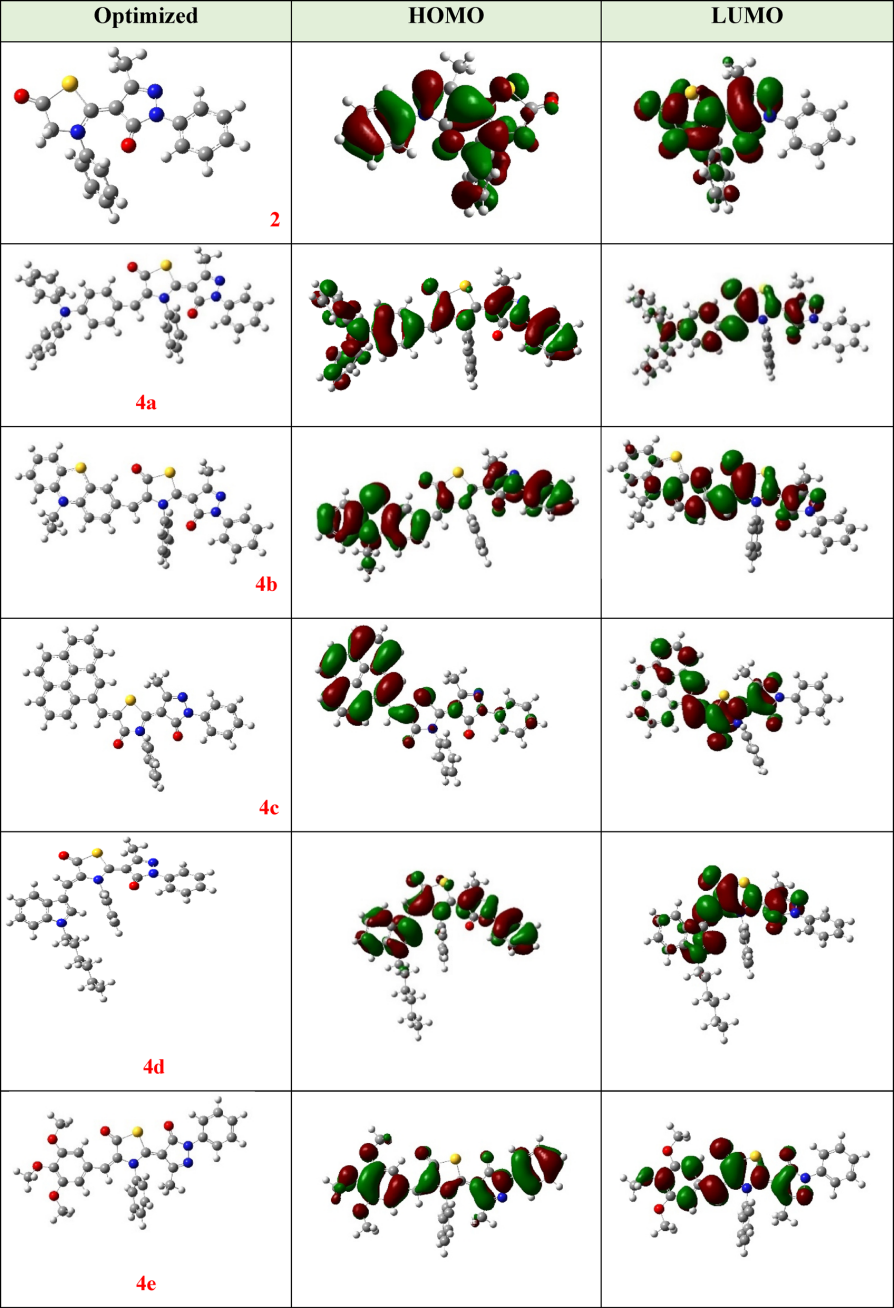

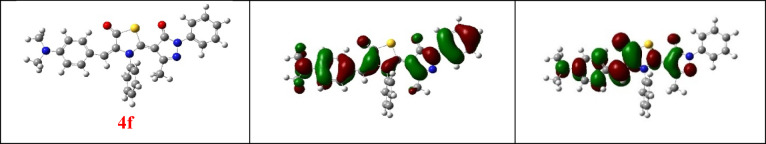
Optimized structures and the frontier molecular orbitals of targeted compounds **2** and **4a-f**.

The DFT theory was performed on investigated thiazolyl-pyrazole hybrids **2** and **4a-f** and computed at B3LYP/6-311G(d, p) by Gaussian 09^43^. Geometry optimization and electron distribution in HOMO and LUMO energy levels are depicted in Fig. 4.

Frontier molecular orbitals are critical properties that explain the reactivity of molecules^44^. The highest occupied molecular orbital (HOMO) refers to electron-donating moieties and is presented in several aromatic aldehydes (triphenylamine, phenothiazine, pyrene, indole, trimethoxy, and dimethylamino-benzene). In contrast, the lowest unoccupied molecular orbital (LUMO) indicates to accept units (pyrazolidine-5-one and thiazolidin-5-one)^45^. As observed in Fig. 4, the electron distribution of the targeted compounds in frontier molecular orbitals is almost similar. Both HOMO and LUMO of compound **2** are detected throughout the whole molecule, except the pyrazolone system’s phenyl ring in LUMO. In the HOMOs of compounds (**4a**, **4b**, **4d**, **4e**, and **4f**), the electrons distribution is located on all molecule exception the thiazolidine-5-one system’s phenyl ring and hexyl alkyl chain of indole unit. While HOMO of derivative **4c** is presented on donor moiety (pyrene ring) only. On the other hand, the LUMOs of all hybrids (**4a-f**) are distributed fundamentally on the thiazole and pyrazole rings these findings show that the reactivity of these compounds is mainly elucidated by the thiazole and pyrazole rings.

Other important factors, such as energy gap (***E***_***g***_), ionization energy (***IP***), electron affinity (***EA***), hardness (***η***), and softness (***s***), describe molecules’ chemical reactivity and biological activity. These factors were computed as follows: Eqs. (1)–(5).

1 $$\:{E}_{g\:}={E}_{HOMO\:}-{E}_{LUMO}$$

2 $$\:IP=-{E}_{HOMO}$$

3 $$\:EA=-{E}_{LUMO}$$

4 $$\:\eta\:=\left(\frac{{E}_{LUMO}-{E}_{HOMO}}{2}\right)$$

5 $$\:s=\frac{1}{\eta\:}$$.

The range of energies of HOMOs and LUMOs were from − 5.05 to -5.69 eV and − 2.23 to -4.72 eV, respectively. Energy gaps (*E*_*g*_) of the investigated hybrids are sorted as **2**(2.46) < **4f**(2.50) < **4e**(2.68) < **4b**(2.73) < **4a**(2.81) < **4d**(2.82) < **4c**(3.32) as shown in Table 1. Moreover, biological activity depends mainly on the ability to transfer electrons from donors to the acceptors in the compound easily^46^. Soft compounds are more reactive than hard compounds because of their ability to donate electrons. The global hardness (*η*) of thiazolyl-pyrazole derivatives **2** and **4a-f** ranged from 1.23 to 1.66 eV. Additionally, the softness values increased in the following sequence: **2** > **4f** > **4e** > **4b** > **4a** > **4d** > **4c**. From the results obtained, it was found that derivative **2** has the lowest energy gap and hardness values and the largest softness values compared to other derivatives. That means this hybrid is the slightest stable kinetically, more reactive, and ideal potent anticancer agent.

**Table 1:** The HOMO-LUMO energies and calculated parameters (eV) of synthesized thiazolyl-pyrazole **2** and **4a-f**.

Cpd. No.	HOMO	LUMO	IP	EA	E	E_g_	η	s
**2**	-5.12	-2.66	5.12	2.66	-2463.923	2.46	1.23	0.81
**4a**	-5.12	-2.31	5.12	2.31	-2047.543	2.81	1.40	0.71
**4b**	-5.23	-2.50	5.23	2.50	-2071.317	2.73	1.36	0.73
**4c**	-5.69	-2.37	5.69	2.37	-1445.863	3.32	1.66	0.60
**4d**	-5.05	-2.23	5.05	2.23	-1839.1111	2.82	1.41	0.70
**4e**	-5.40	-2.72	5.40	2.72	-2087.2764	2.68	1.34	0.74
**4f**	-5.07	-2.57	5.07	2.57	-2220.4810	2.50	1.25	0.80

#### ADME prediction

A complete investigation of physicochemical parameters for different conjugates **2** and **4a-f** was introduced in Table 2 using the Swiss ADME tool. Lipinski’s rule “rule of five” helps define the best drug taken orally^47^. Lipinski’s rule is based on the different factors; molecular weight (MW ≤ 500), lipophilicity (Mlog *P* ≤ 4.15), the number of hydrogen bond acceptors (HBA ≤ 10), the number of hydrogen bond donors (HBD ≤ 5), and the number of violations (*n*Vs ≤ 1)^48^. We found that derivatives **2**, **4d**, **4e**, and **4f** apply the parameters of this rule, while derivatives **4a**, **4b**, and **4c** do not apply due to having MW exceed 500 and **MLogP** overcome 4.15. So, these derivatives **2**, **4d**, **4e**, and **4f** have the more attainable drug properties. Moreover, the flexibility of the molecule was determined by the number of rotatable bonds, the molecule with nRB less than 10 is flexible and has good oral bioavailability^49^. All analogs achieved this requirement. Additionally, compounds **2**, **4d**, **4e**, and **4f** have ideal value of BS **=** 0.55%. Topological polar surface area (TPSA) shows the molecule’s ability to interact with membranes, molecules with high TPSA values have problems with permeability^50^. On the other hand, the pharmacokinetic features such as gastrointestinal absorption (GI) and the blood-brain barrier (BBB) were discussed, all hybrids exhibited high GI absorption and non-permeating BBB.

**Table 2:** Predicted physicochemical and Pharmacokinetic characteristics of thiazolyl-pyrazole derivatives **2** and **4a-f**.

Factors	Cpds.						
	**2**	**4a**	**4b**	**4c**	**4d**	**4e**	**4f**
M.W	349.41	604.72	586.73	561.65	560.71	527.59	480.58
HBD	0	0	0	0	0	0	0
HBA	3	3	3	3	3	6	3
MLogP	2.13	5.04	4.78	4.66	4.04	2.18	3.04
nVs	0	2	2	2	1	1	0
nRB	2	6	4	3	8	6	4
TPSA (Å²)	78.28	86.15	111.45	82.91	87.84	110.60	86.15
Solubility	Moderate	Poor	Poor	Poor	Poor	Poor	Poor
GI absorption	High	High	High	High	High	High	High
BBB	No	No	No	No	No	No	No
BS	0.55	0.17	0.17	0.17	0.55	0.55	0.55
Lipinski’s Violation	Yes	No	No	No	Yes	Yes	Yes

**Fig. 5:**
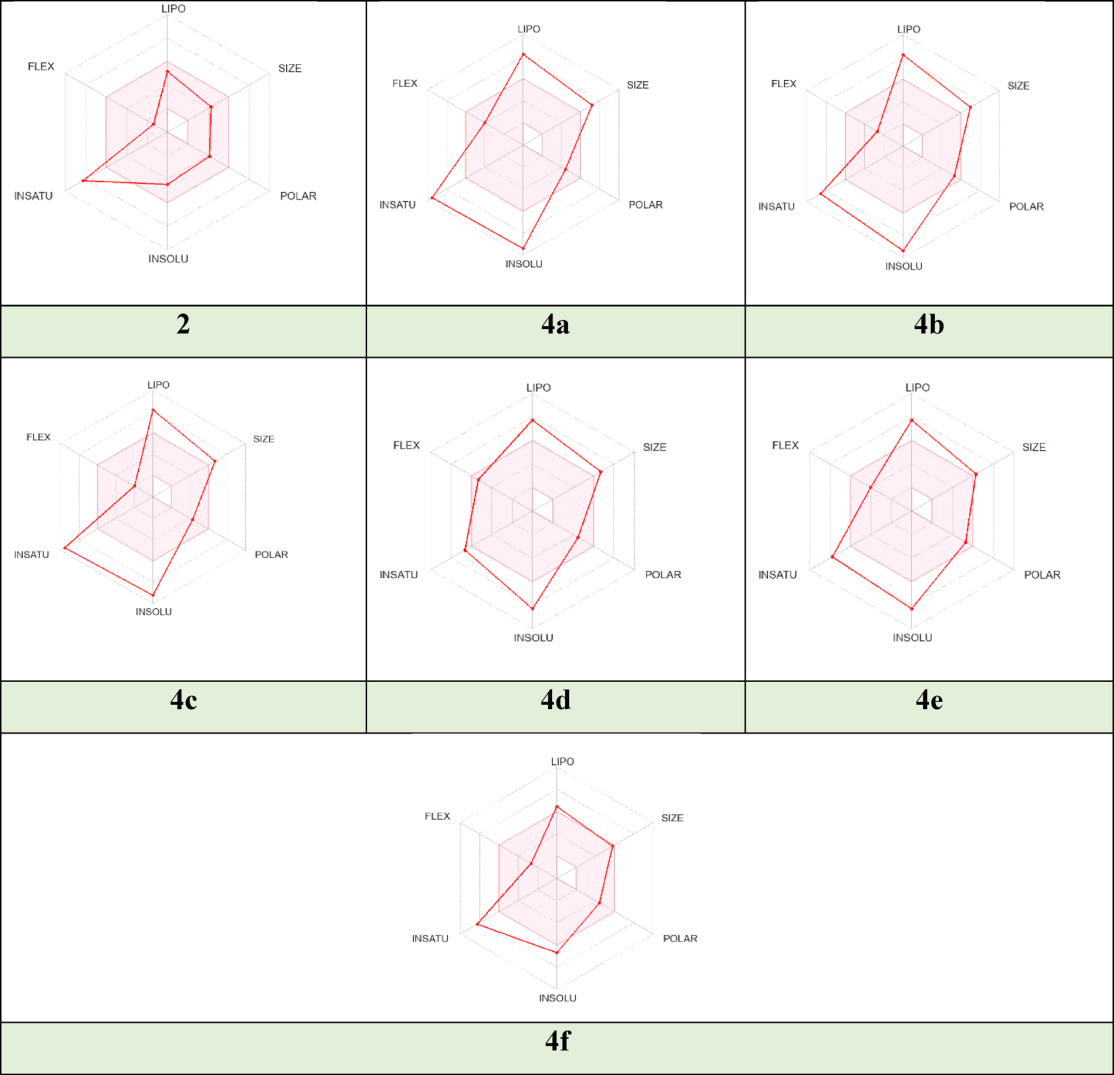
The bioavailability radar chart of target compounds **2** and **4a-f**.

The radar charts demonstrate an evaluation of basic physicochemical factors such as lipophilicity (**LIPO**), polarity (**POLAR**), size (**SIZE**), unsaturation (**INSATU**), solubility (**INSOLU**), and flexibility (**FLEX**). The molecule must be depicted in a pink area to be considered a drug^51^. The most potent compounds **2** and **4f** have an optimal range of all properties except INSATU for compound **2** and LIPO, INSOLU, and INSATU for compound **4f**, respectively. In contrast, compounds **4a-e** have only two factors lying in the pink zone: FLEX and POLAR, as illustrated in Fig. 5.

**Fig. 6:**
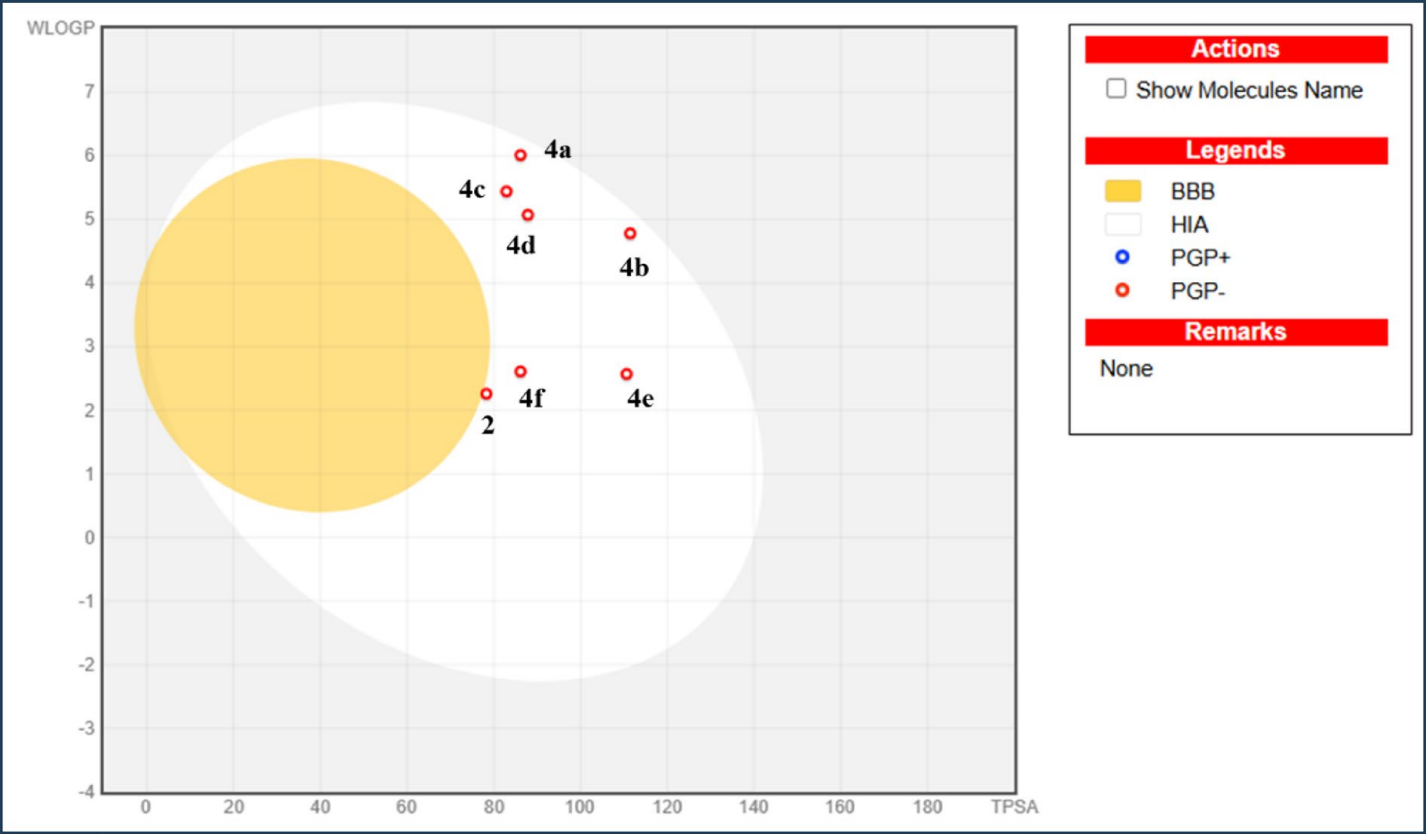
Boiled-egg chart of thiazolyl-pyrazole derivatives **2** and **4a-f**.

BOILED-Egg model calculates lipophilicity and polarity, which is critical for investigating GI absorption and BBB access, and that helps in drug discovery^52^. All derivatives located in the white zone and absorbed by the gastrointestinal and presented with red dots in Fig. 6 which means compounds are not outflowed from CNS by PGP-. According to the results above, hybrid **2** outperformed the other hybrids, it revealed the lowest values of M.W and MLogP (349.41 and 2.13), respectively so that it recorded moderate solubility. This compound has a high ability to interact with membranes. It permeated from HIA and became the nearest derivative to BBB, which is attributed to low TPSA value (78.28 Å²).

#### PPI network and hub targets

**Fig. 7:**
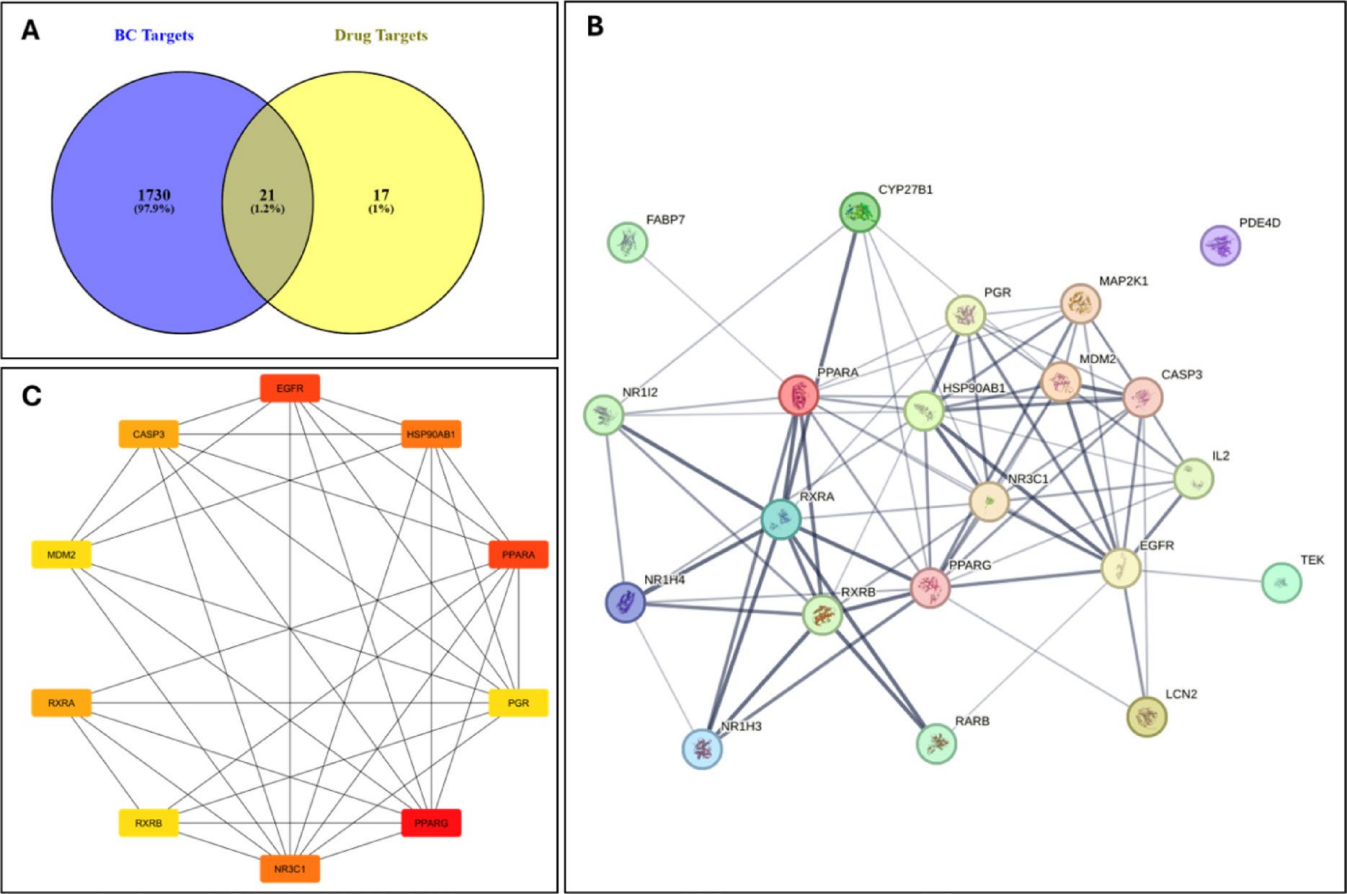
Drug-breast cancer targets. (**A**) Venn diagram of the drug and disease targets. The intersection represents the potential target of the drug in the treatment of breast cancer. (**B**) The original PPI network from the STRING database. **C**) Top ten hub genes were calculated using the CytoHubba tool and the degree algorithm and ranked by color coding. The higher the rank, the darker the red color.

The molecular targets of the tested compound were identified by PharmMapper server. After filtration, 38 targets had normalized Fit scores and Z-scores greater than 0.5 as illustrated in Fig. 7A. Protein-protein interaction network for the common targets between the tested compound and breast cancer were constructed by STRING database. Figure 7B displays the 21 nodes and 76 edges that were collected. Each edge in the network represents a protein-protein interaction. The network illustrated in confidence view where the line thickness represents the degree of data support. The top 10 hub genes calculated by CytoHubba and ordered according to the Degree algorithm are mentioned in Fig. 7C. These hub genes include PPARG, EGFR, and PPARA, Nuclear receptor subfamily 3 group C member 1(NR3C1), Heat shock protein 90 alpha family class B member 1 (HSP90AB1), Retinoid X receptor alpha (RXRA), Caspase 3 (CASP3), Progesterone receptor (PRG), Mouse double minute 2 homolog (MDM2) and Retinoid X receptor beta (RXRB). The top 3 most significant hub targets PPARG, EGFR, and PPARA (had the same rank as EGFR) were selected for molecular docking studies to assess their affinities and interaction modes with the tested compound.

### Anticancer study

The in vitro cytotoxicity of the tested compounds was estimated using the MTT assay toward MCF-7 and MDA-MB-231 breast cancer cells. The IC₅₀ values are expressed in µM and are presented in Table 3.

**Table 3:** Cytotoxicity results of newly thiazolyl-pyrazole conjugates **2** and **4a-f**.

Cpd. no.	In vitro cytotoxicity IC_50_ ± S. D (µM)	
	MCF-7	MDA-MB-231
2	69.63 ± 1.07	22.84 ± 0.73
4a	98.81 ± 0.98	93.02 ± 0.80
4b	89.25 ± 0.88	86.81 ± 2.32
4c	> 100	> 100
4d	> 100	> 100
4e	84.25 ± 1.67	79.21 ± 0.69
4f	82.34 ± 2.01	75.58 ± 0.54

**Fig. 8:**
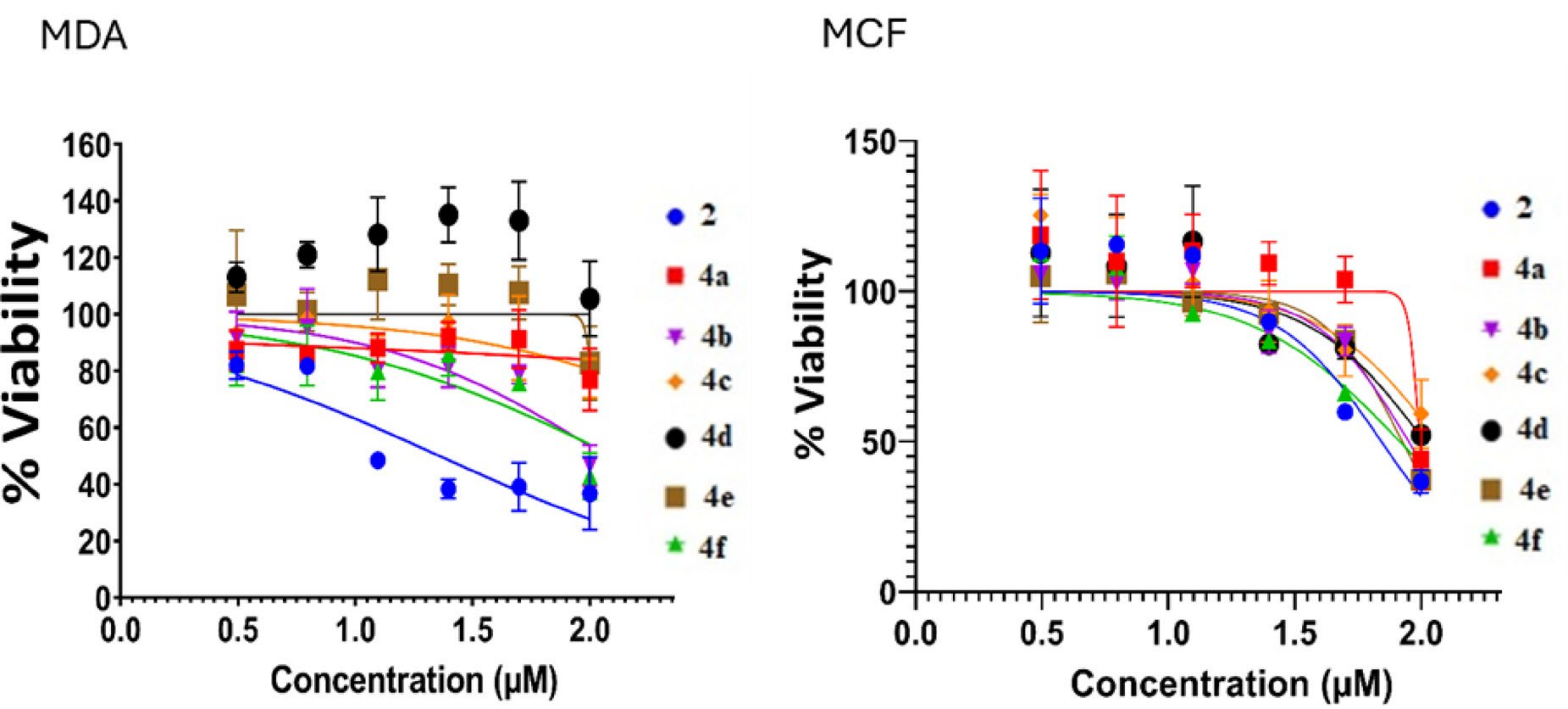
The cell viability of breast cancer cells with compounds **2** and **4a-f**.

Figure 8 shown MCF-7 and MDA-MB-231 cell lines viability are sensitive to compounds **2**, **4f**, and **4e** in the presence of varying concentrations. Thiazolyl-pyrazole **2** demonstrated the highest activity, with IC₅₀ values of 69.63 ± 1.07 µM for MCF-7 and 22.84 ± 0.73 µM for MDA-MB-231. conjugates **4a** and **4b** exhibited weak cytotoxicity, with IC₅₀ values of 98.81 ± 0.98 µM and 93.02 ± 0.80 µM for **4a**, and 89.25 ± 0.88 µM and 86.81 ± 2.32 µM for **4b** against MCF-7 and MDA-MB-231 cells, respectively. derivatives **4c** and **4d** showed negligible cytotoxicity. Otherwise, derivatives **4e** and **4f** displayed moderate cytotoxicity, with **4e** yielding IC₅₀ values of 84.25 ± 1.67 µM and 79.21 ± 0.69 µM, while **4f** exhibited 82.34 ± 2.01 µM and 75.58 ± 0.54 µM for MCF-7 and MDA-MB-231, respectively. Among all tested compounds, compound **2** showed the highest cytotoxic effect, particularly against MDA-MB-231 cells, hence compound **2** effects on MDA-MB-231 was subjected for further studies.

#### Structure-activity relationship (SAR)

**Fig. 9:**
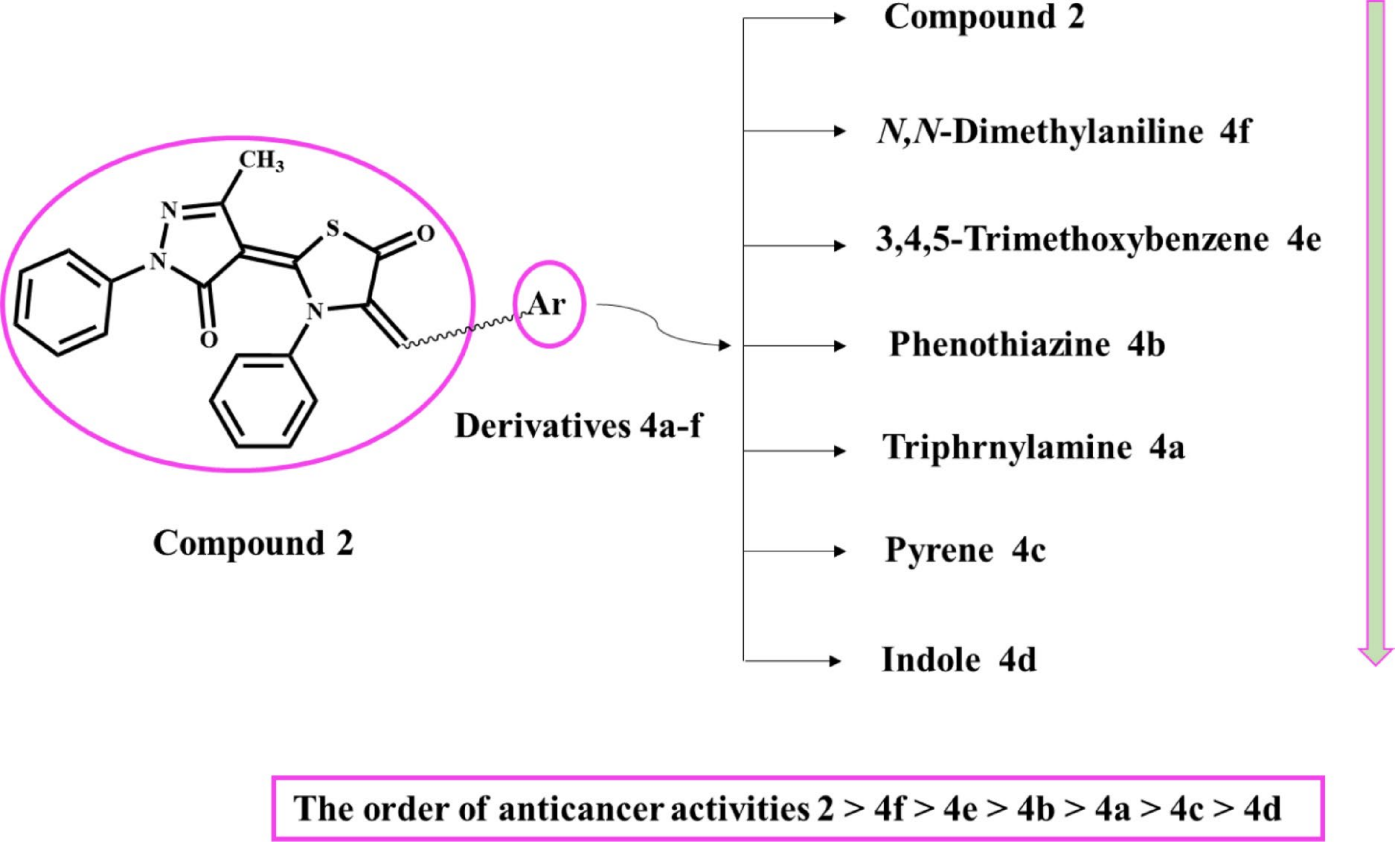
The SAR survey tested compounds **2** and **4a-f**.

As can be seen in Figs. 2 and 3, the starting molecule and six derivatives have been synthesized, differing in the substituents on the active methylene group of the thiazolidin-5-one ring. The SAR survey of compounds **2** and **4a-f** as breast anticancer agents is schematically presented in Fig. 9. Starting compound 2, without any substitution on the active methylene group, exhibited eminent IC_50_ values toward breast cancer cells, MCF-7 and MDA-MB-231 (69.63 and 22.84 µM, respectively) in comparison to six hybrids **4a-f** with a range of IC_50_ (75.58–98.81 µM) due to present various heterocyclic rings, pyrazole and thiazole. Hybrid **4f**, with *N*,* N*-dimethylaniline substitution, displayed good inhibition (IC_50_ = 82.34 and 75.58 µM) compared to other derivatives **4a-e**. On the other hand, comparing the activities of hybrids **4e**(IC_50_ = 84.25 and 79.21 µM) and **4b**(IC_50_ = 89.25 and 86.81 µM) revealed that the replacement of 3,4,5-trimethoxybenzene unit with phenothiazine unit in compound **5b** led to a slight decline in inhibitory strength. Furthermore, changing the substitution to triphenylamine, as in compound **4a**(IC_50_ = 98.81 and 93.02 µM), decreased the activity. The last two compounds of this series, compound **4c** with large aromaticity part (pyrene) and compound **4d** containing indole with non-polar alkyl chain (hexyl chain) demonstrated the lowest potent toward breast anticancer agents. From the above discussion, it was found modification of the starting compound **2** by condensation with different aldehydes inhibited activity against two cell lines.

#### Compound 2 inhibited wound healing

**Fig. 10:**
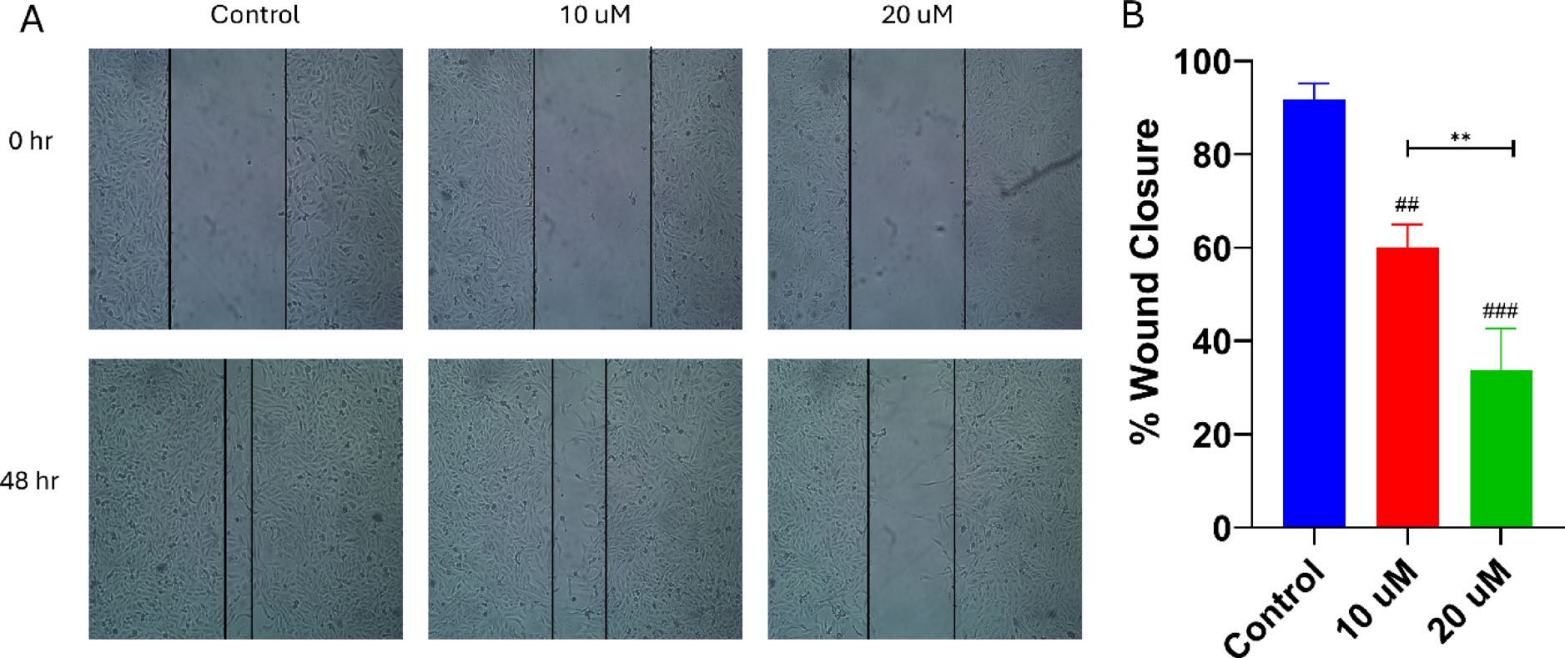
Effect of compound **2** on migration of MDA-MB-231 cells: (**A**) Wound area after 48 h of control and treated cells with 10 µM and 20 µM. (**B**) Bar plots for the migration rates of control and treated cells.

The wound healing assay was performed to assess the anti-migratory effects of compound **2** on MDA-MB-231 cells at 10 µM and 20 µM concentrations. The results demonstrated that both doses significantly inhibited wound closure compared to the control. Moreover, a dose-dependent effect was observed, as 20 µM exhibited significantly greater wound inhibition than 10 µM (Fig. 10).

#### Molecular Docking study

**Fig. 11:**
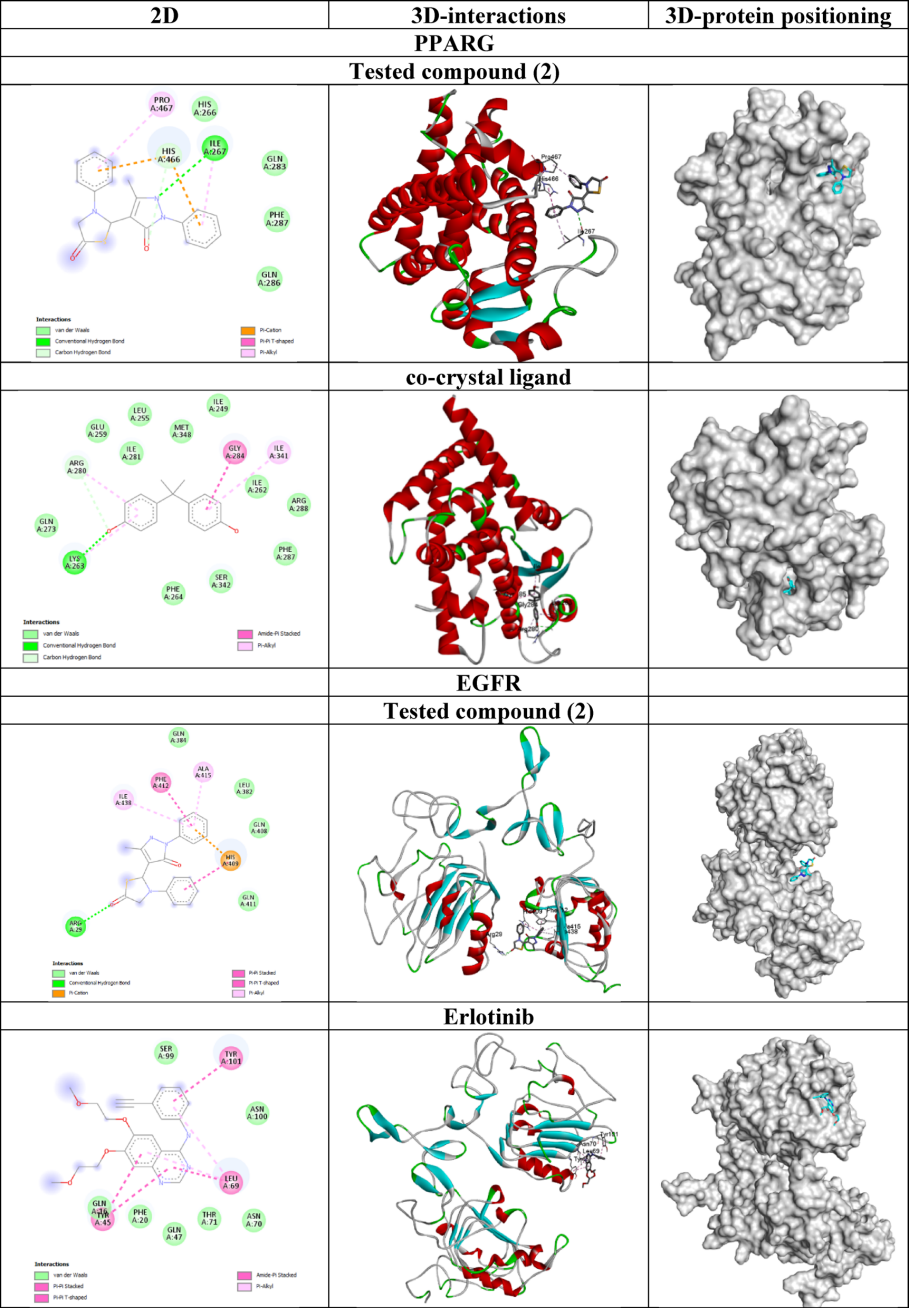

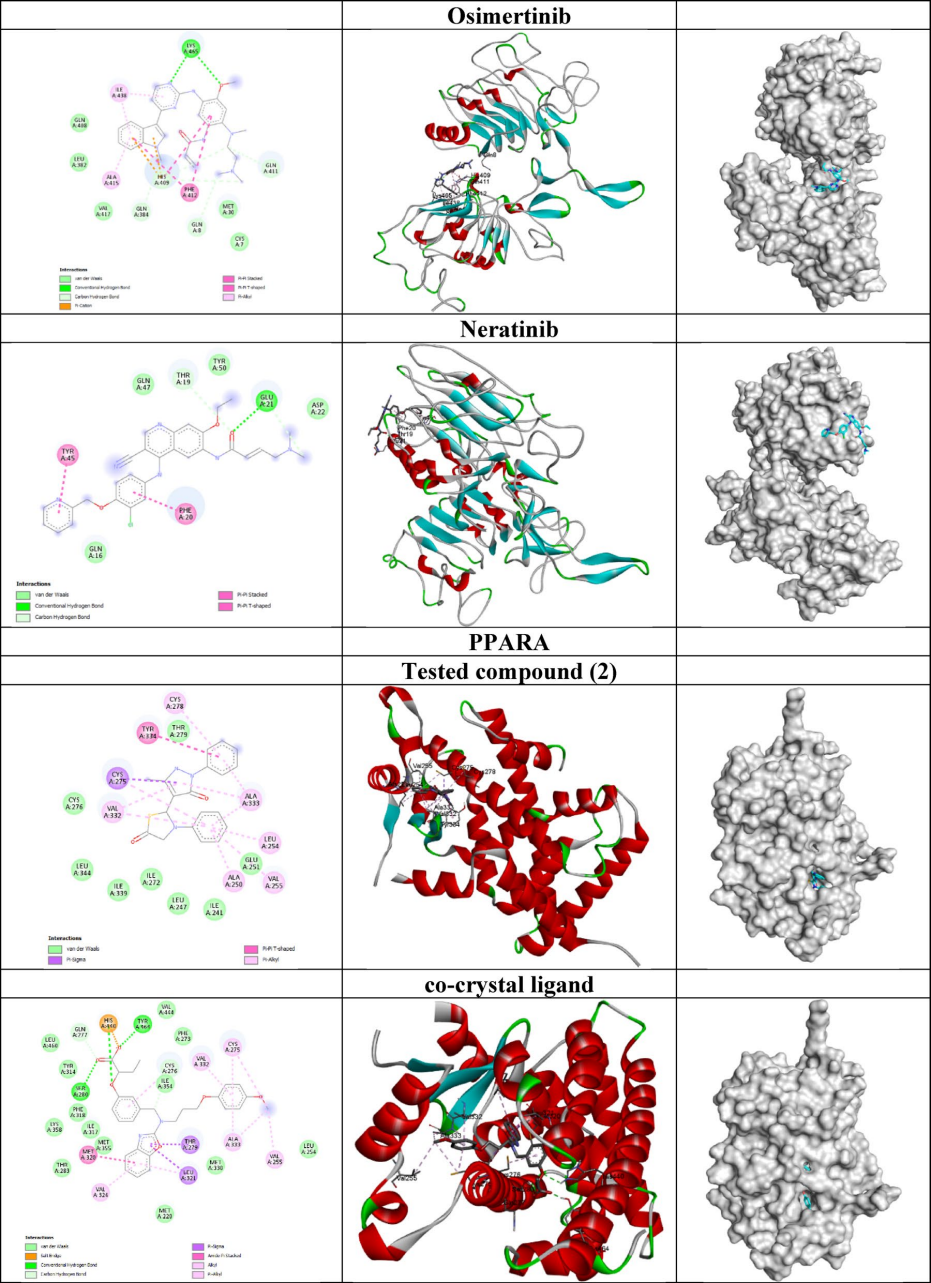
The 2D and 3D structures of the docked complexes.

The molecular docking results by GNINA software revealed insights into the binding affinities and predicted interactions between the tested compound and reference ligands with their respective targets: PPARG, EGFR, and PPARA as shown in Table 4; Fig. 11. The CNN pose score reflects the confidence of the convolutional neural network (CNN) in predicting the correctness of the ligand’s pose.

**PPARG** is a key factor in the regulation of lipid metabolism and energy homeostasis. It is an important therapeutic target in breast cancer^53,54^. The tested compound exhibited a moderate binding affinity of -4.53 kcal/mol for PPARG, which is weaker than that of the co-crystallized ligand (-6.67 kcal/mol). Similarly, the CNN affinity and pose scores for the tested compound (4.946 and 0.592, respectively) were also lower than those of the co-crystallized ligand (5.571 and 0.799), suggesting that the tested compound may not bind as effectively or in the same manner as the co-crystallized ligand. Redocking of the co-crystallized ligand generated the best pose of the highest affinity and RMSD of 1.854 Å which indicates that the predicted pose by GNINA software is very close to that in the original crystal confirming the reliability of the docking protocol.

**EGFR** is overexpressed in breast cancer and is crucial in regulating and sustaining key biological features including stemness^55^proliferation^56^as well as invasion and metastasis^57^. The tested compound demonstrated a binding affinity of -6.53 kcal/mol for EGFR, which is almost similar to that of the standard drug Erlotinib (-6.81 kcal/mol) but weaker compared to Osimertinib (-7.68 kcal/mol) and Neratinib (-9.11 kcal/mol). The CNN affinity of the tested compound 5.059 was also slightly lower than that of Erlotinib (5.697) and Neratinib (6.769), indicating that the tested compound may not achieve the same level of interaction as the more potent reference ligands. The interaction between the tested drug and EGFR was stabilized by a hydrogen bond with Arg29 residue, *π*-cation and *π*-*π* stacking interactions with His409 residue, *π*-alkyl interactions with Ala415 and Ile438 residues in addition to van der Waal interactions. The CNN pose score of the tested compound 0.810 reflected the confidence of the CNN model for the correctness of the predicted docking pose.

**PPARA** regulates fatty acid homeostasis, influences cell cycle and apoptosis in normal and tumor cells, and so was considered as a therapeutic target in breast cancer^58^. The tested compound showed a binding affinity of -7.11 kcal/mol towards PPARA, which was significantly weaker than the co-crystallized ligand (-12.63 kcal/mol). The CNN affinity for the tested compound (5.979) was also notably lower than that of the co-crystallized ligand (7.241) which highlights a substantial gap in binding performance.

While the docking scores of the tested compound with PPARA protein were better than those with EGFR, the EGFR-drug complex has the highest CNN score and hence selected for further studies.

**Table 4:** Molecular Docking results of hybrid **2** and the co-crystallized ligands with PPARG, EGFR, and PPARA genes.

Compound	Affinity (kcal/mol)	CNN affinity	CNN pose score
PPARG			
Tested compound (2)	-4.53	4.946	0.592
co-crystal ligand	-6.67	5.571	0.799
EGFR			
Tested compound (2)	-6.53	5.059	0.810
Erlotinib	-6.81	5.697	0.837
Osimertinib	-7.68	6.365	0.654
Neratinib	-9.11	6.769	0.827
PPARA			
Tested compound (2)	-7.11	5.979	0.343
co-crystal ligand	-12.63	7.241	0.895

### Molecular dynamic simulation (MDS) study

To further assess and gain deeper insights into the interaction between the tested compound and EGFR protein, the docked complex was subjected to 100 ns MD simulation under conditions mimicking a real-life physiological environment.

**Fig. 12:**
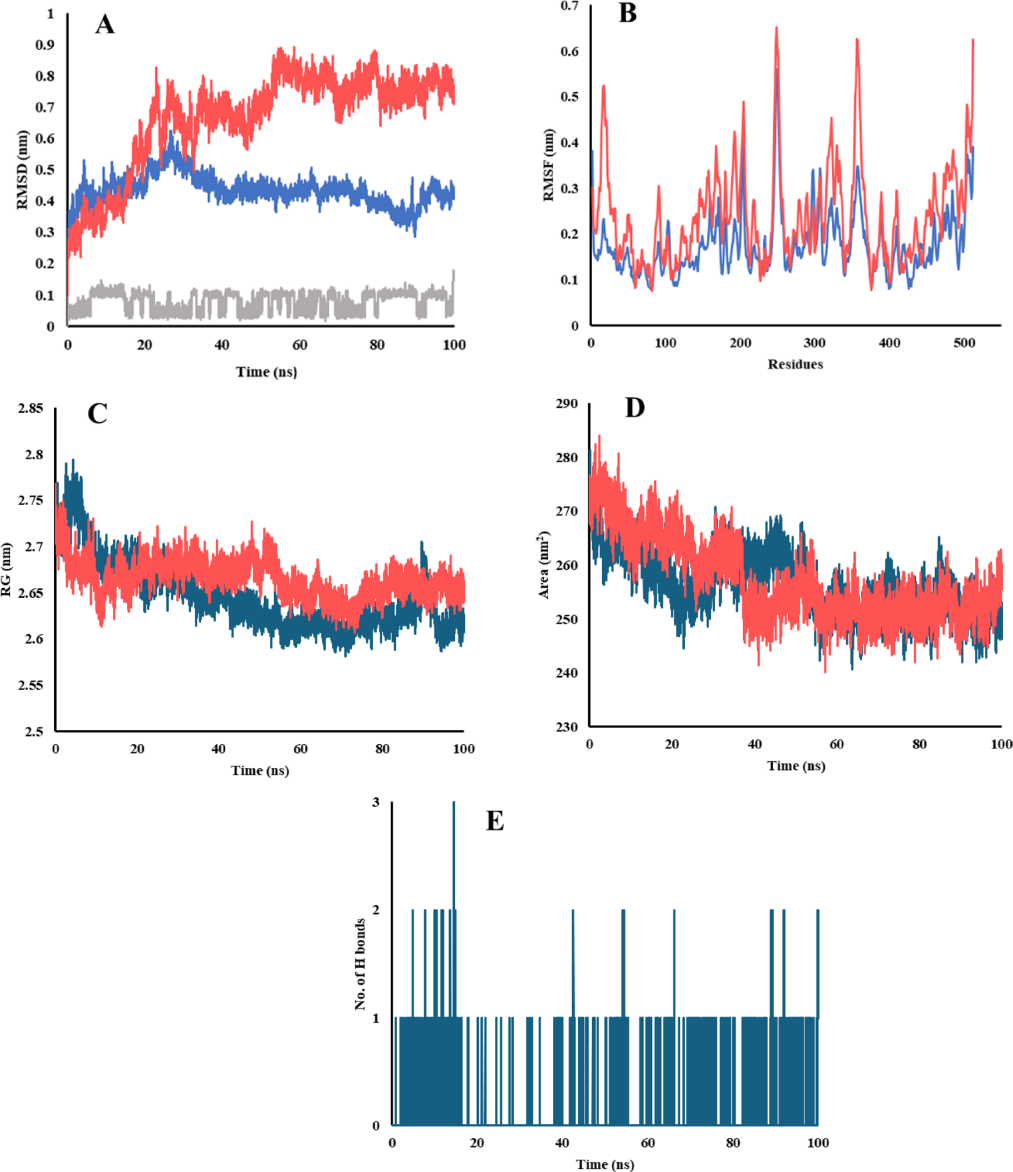
MD simulation results of the EGFR-drug complex after 100 ns simulation, (**A**) RMSD plot, (**B**) RMSF plot, (**C**) RG plot, (**D**) SASA plot, and (**E**) H.B plot, complex (in blue), protein (in red), and ligand (in gray).

Root Mean Square Deviation (RMSD) analysis revealed insights about the stability and conformational changes of the protein alone or in complex with the tested compound. The complex exhibited a notable lower RMSD values with average 0.43 ± 0.05 nm compared to the unbound protein with average 0.66 ± 0.16 nm (Fig. 12A). The protein alone showed an initial increase in RMSD reaching 0.81 nm at 23 ns followed by a slight stabilization with notable fluctuations till the end of the simulation. On the other hand, the complex showed a stable RMSD profile with a slight plateau around 27 ns. This difference in RMSD profiles reflects the structural stability induced by the ligand binding. The calculated average RMSD of the ligand was 0.08 ± 0.03 nm reflects the stability of the compound in the binding pocket of EGFR protein.

Root Mean Square Fluctuation (RMSF) was calculated to estimate the fluctuations and flexibility of the protein residues (Fig. 12B). The unbound EGFR protein exhibited generally higher fluctuations with an average RMSF of 0.24 ± 0.10 nm. This higher RMSF indicates more structural flexibility in those regions when unbound. In contrast, the complexed EGFR showed significantly lower fluctuations (average 0.17 ± 0.07 nm) in many regions specifically 10–30, 320–338, and 350–370, indicating restrictions in the movement of these regions, which might play a role in maintaining structural integrity or functional interactions.

Radius of gyration (RG) analysis provided information about the compactness of EGFR protein by calculating the root mean square distance of its atoms from the protein’s center of mass. From the analysis, the RG of the complex was generally slightly lower (average 2.64 ± 0.04 nm) than the unbound protein (average 2.66 ± 0.02 nm) as illustrated in Fig. 12C. These results indicated that ligand binding induced slight structural changes in the protein leading to a more compact structure which may affect the protein functions.

Solvent-accessible surface area (SASA) analysis provided insights into the surface area of EGFR that is accessible to solvent (water) molecules. The unbound EGFR protein exhibited an average SASA of 257 ± 7.56 nm^2^ (Fig. 12D). Ligand binding did not significantly affect the SASA of the protein, as the complex exhibited an average SASA of 256 ± 5.75 nm^2^.

Hydrogen bonds play a key role in stabilizing complex formation. Hydrogen bonds formed between the drug and EGFR protein during the 100 ns MD simulation were analyzed and represented in Fig. 12E. The analysis revealed that for the majority of the simulation time, one hydrogen bond was observed, with occasional increases to two or three bonds (at 14 ns), suggesting transient binding events. Periods of no hydrogen bonds were also evident, indicating dynamic interaction patterns.

### Compound 2 inhibited EGFR

**Fig. 13:**
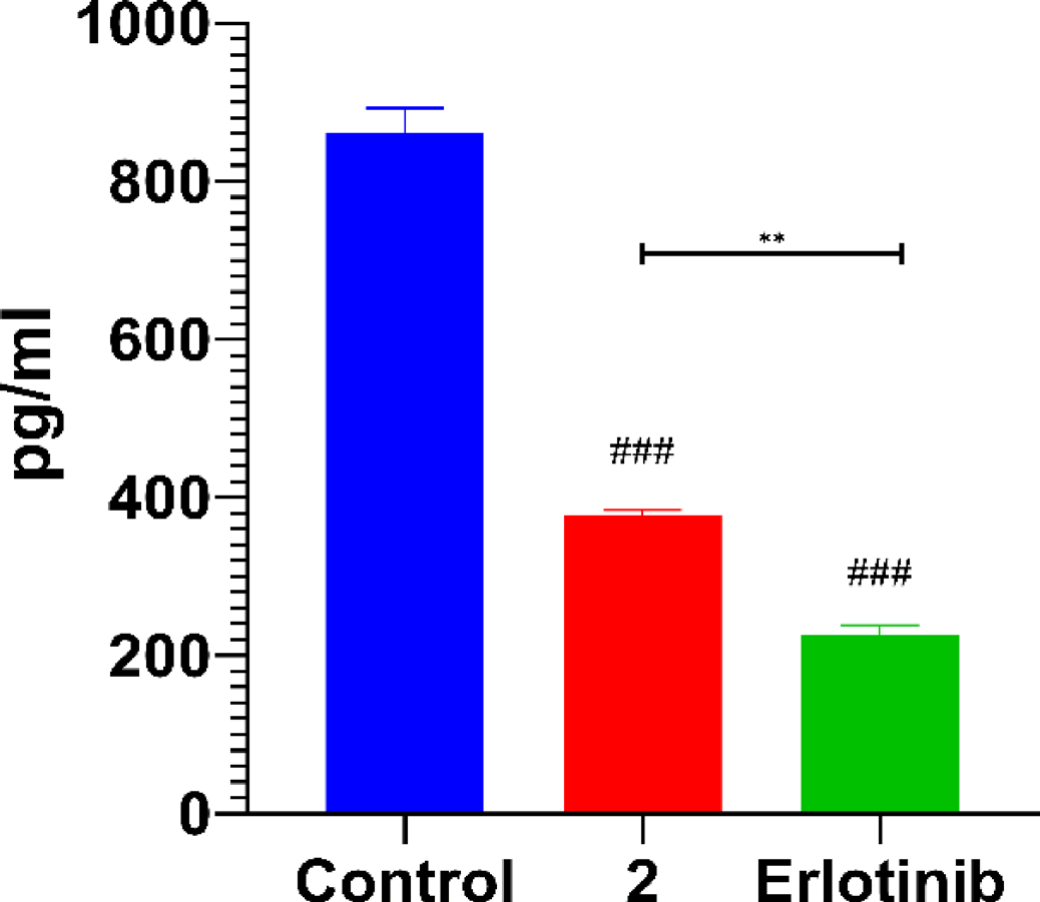
Bar plot for the EGFR expression of control, compound **2**, and erlotinib.

The ELISA assay was conducted to validate the inhibitory effect of thiazolyl-pyrazole **2** on EGFR expression. The results revealed that conjugated **2** significantly inhibited EGFR levels, confirming its potential as an EGFR-targeting agent. When compared to erlotinib, a known EGFR inhibitor, compound **2** exhibited a comparable inhibitory effect, though its suppression of EGFR expression was significantly lower than that of erlotinib as seen in Fig. 13.

## Conclusion

A new starting compound **2** and six new conjugates **4a–f** containing a thiazolyl-pyrazole moiety were successfully synthesized. The optimized structure and electronic parameters of these innovative scaffolds were evaluated using DFT computational methods. The antitumor reactivity revealed that these conjugates have a broad range of action against two breast cancer cells, MCF-7 and MDA-MB-231, where hybrid **2** demonstrated the highest efficiency with IC_50_ values of 69.63 and 22.84 µM, respectively. Additionally, the mechanism of anticancer activity for the most potent thiazolyl-pyrazole **2** was explained *via* wound healing technique on the MDA-MB-231 cancer cell and recorded a significant cell migration rate compared to control. To elucidate the molecular targets of the newly synthesized compound **2**, PharmMapper server and protein-protein interaction network were studied. In silico, molecular docking studies were done to investigate the ability of the derivative **2** to potently inhibit **PPARG**, **EGFR**, and **PPARA** proteins. Hybrid **2** showed remarkable CNN affinity and different interactions with **EGFR** inhibitor. This result was validated with molecular dynamics and ELISA assay theoretically and experimentally, respectively.

## Electronic supplementary material

Below is the link to the electronic supplementary material.

Supplementary Material 1

## Data Availability

No datasets were generated or analysed during the current study.
