# Supplementary material for: Discovery and molecular characterization of a potent thiazolyl-pyrazole hybrid targeting EGFR for breast cancer therapy
Source: Sci Rep. 2025 Jul 3;15:23743. doi: 10.1038/s41598-025-07261-6 (PMC12229662; doi:10.1038/s41598-025-07261-6)
Supplement: Supplementary file 1 — Supplementary Material 1 [file 41598_2025_7261_MOESM1_ESM.docx]

**Discovery and Molecular Characterization of a Potent Thiazolyl-Pyrazole Hybrid Targeting EGFR for Breast Cancer Therapy**

Samar E. Mahmoud, Ahmed A. Fadda, Ehab Abdel-Latif & Mohamed R. Elmorsy^🖂^

*Department of Chemistry, Faculty of Science, Mansoura University, 35516 Mansoura, Egypt.*

^🖂^ *Correspondence:* [*m.r.elmorsy@gmail.com*](mailto:m.r.elmorsy@gmail.com) *& m.r.elmorsy@mans.edu.eg*

1. **Spectral analysis:**


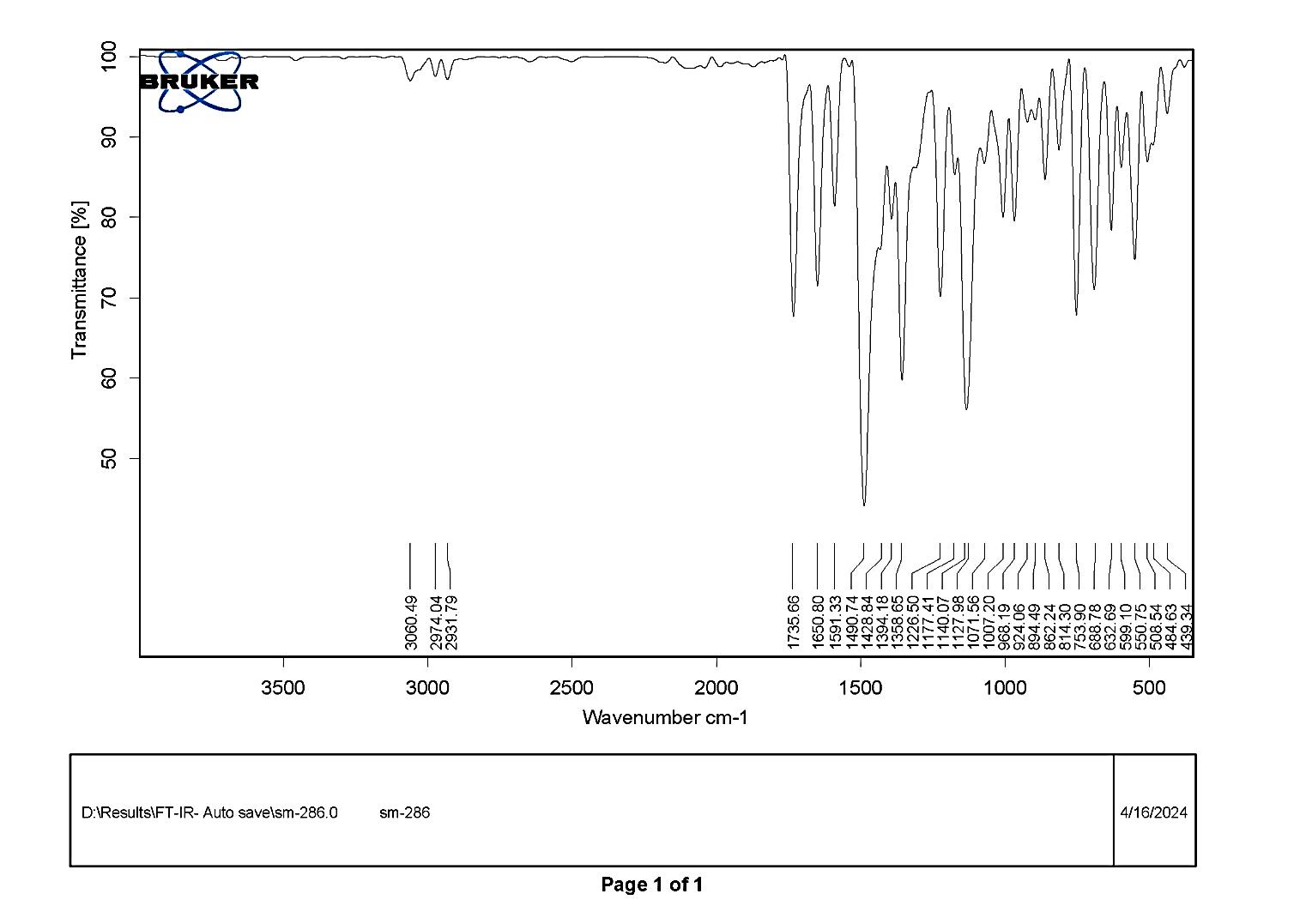

**Figure (S1): IR spectrum of compound 2.**

**
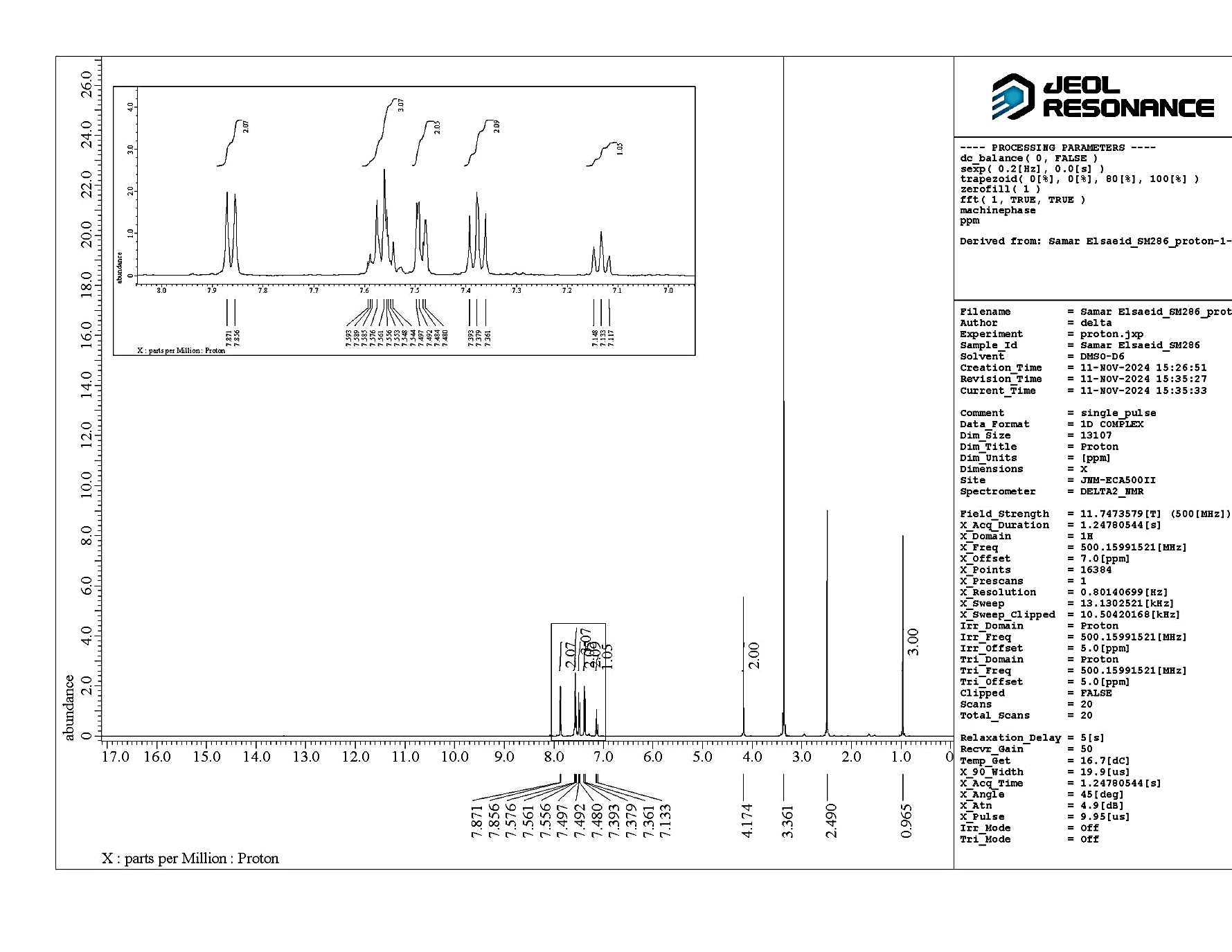
**

**Figure (S2): ^1^H NMR spectrum of compound 2.**

**
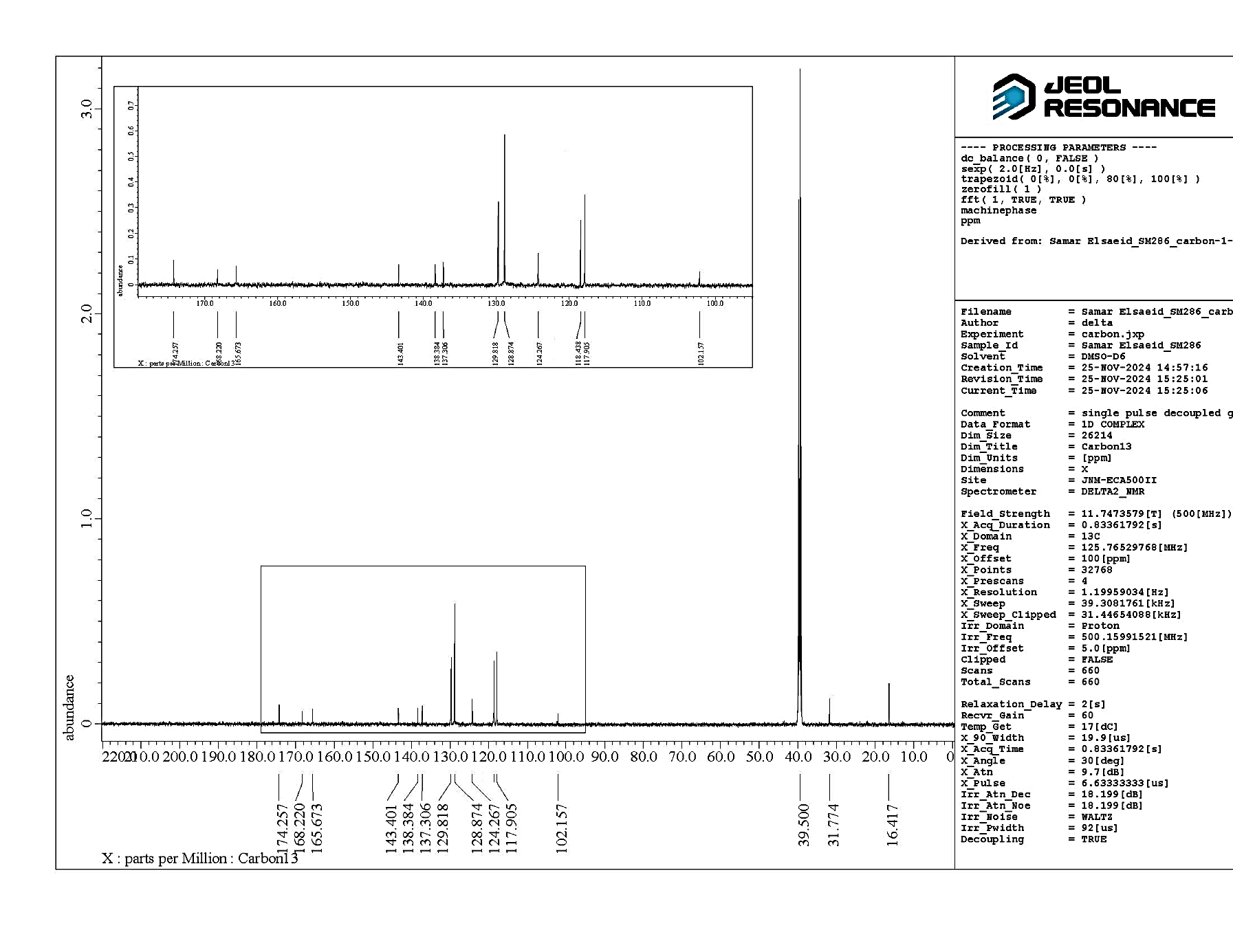
**

**Figure (S3): ^13^C NMR spectrum of compound 2.**


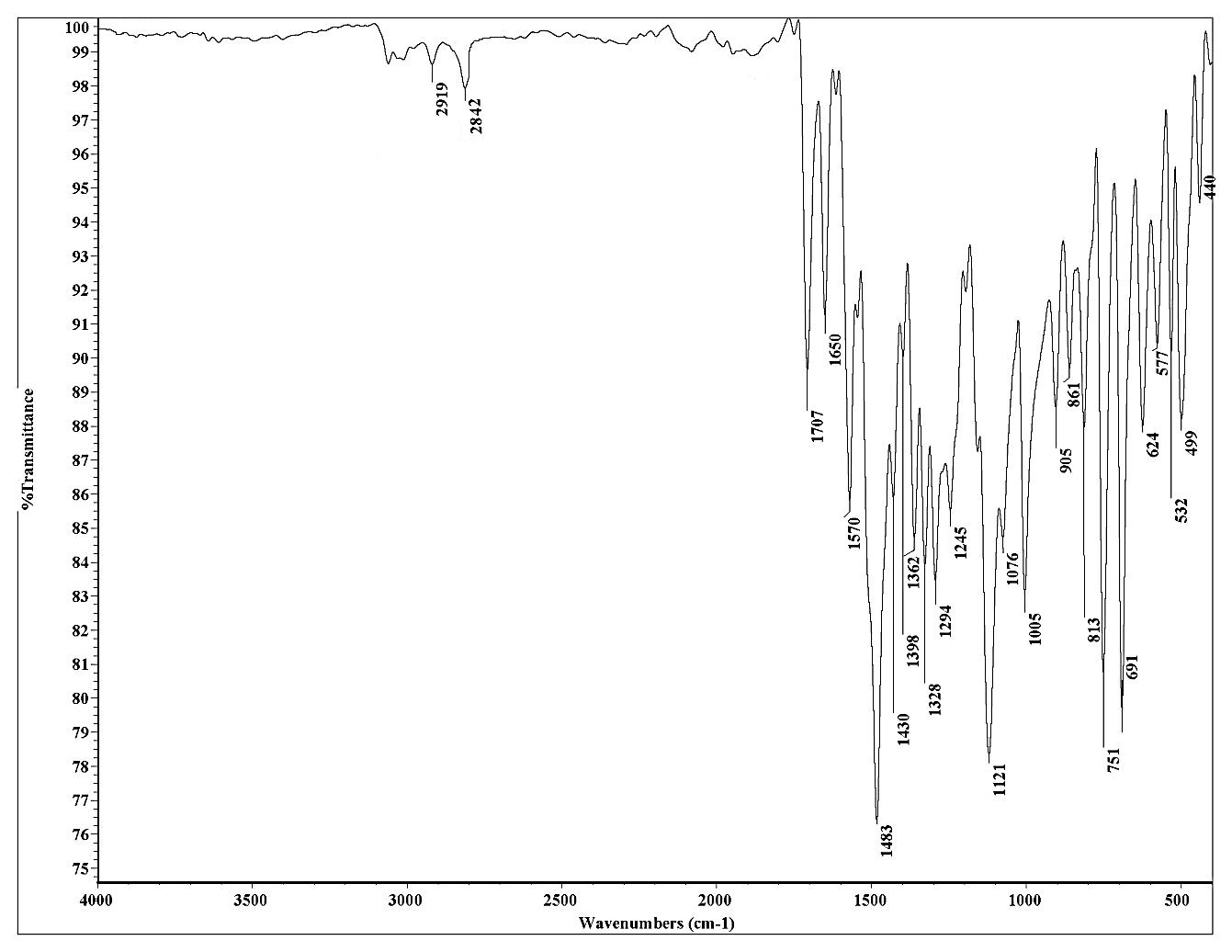


**Figure (S4): IR spectrum of compound 4a.**


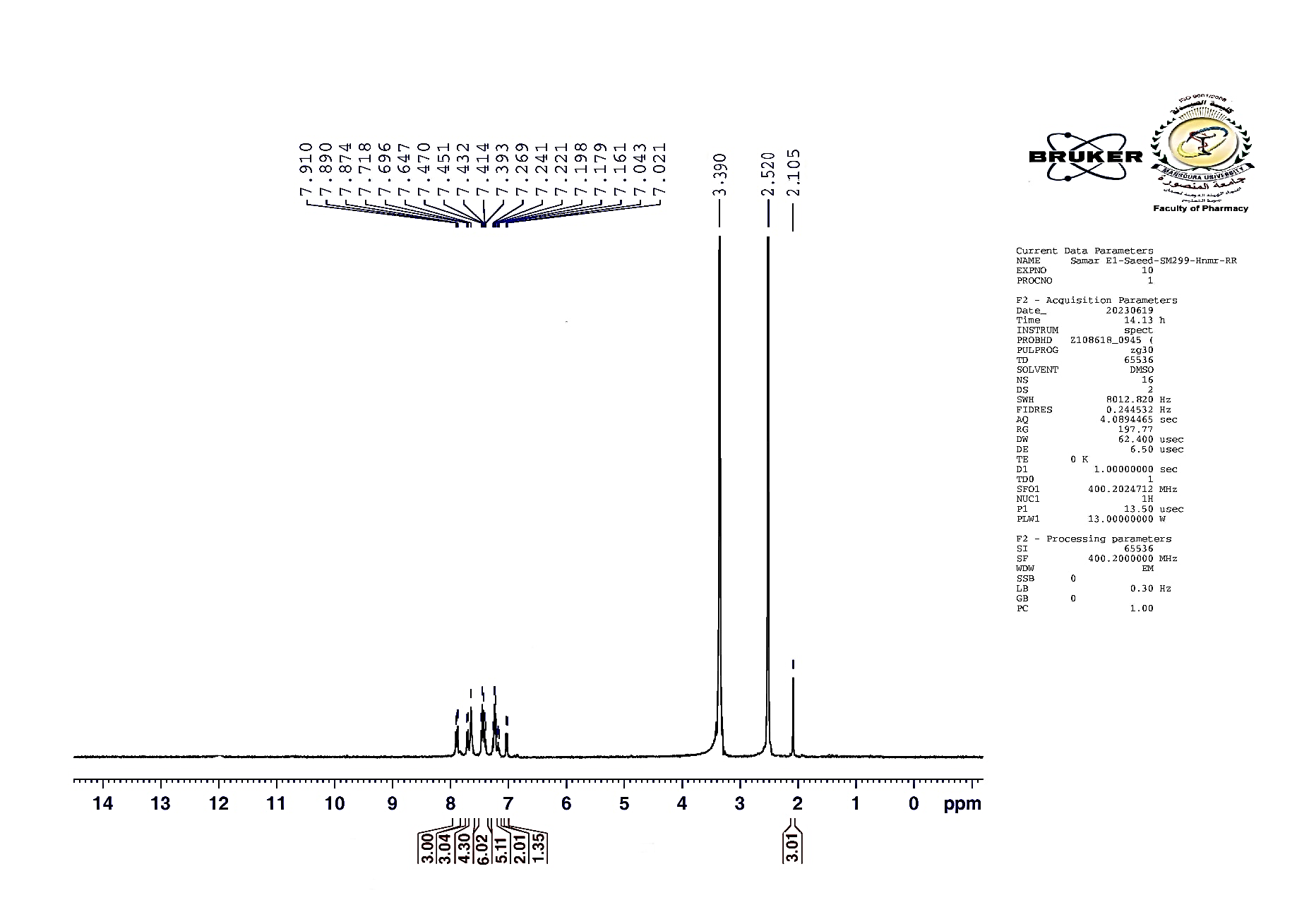

**Figure (S5): ^1^H NMR spectrum of compound 4a.**


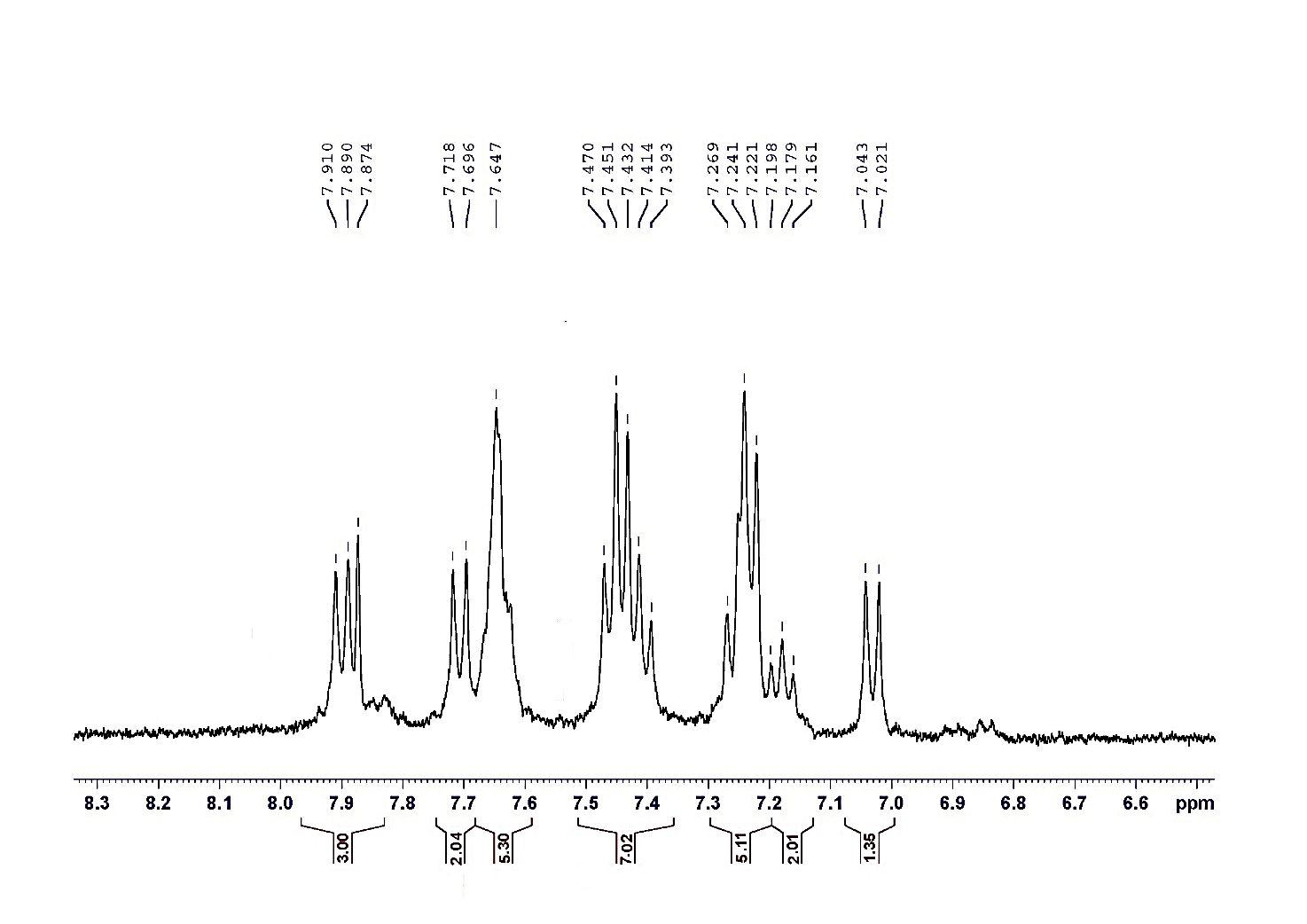

**Figure (S6): ^1^H NMR spectrum of compound 4a.**

**
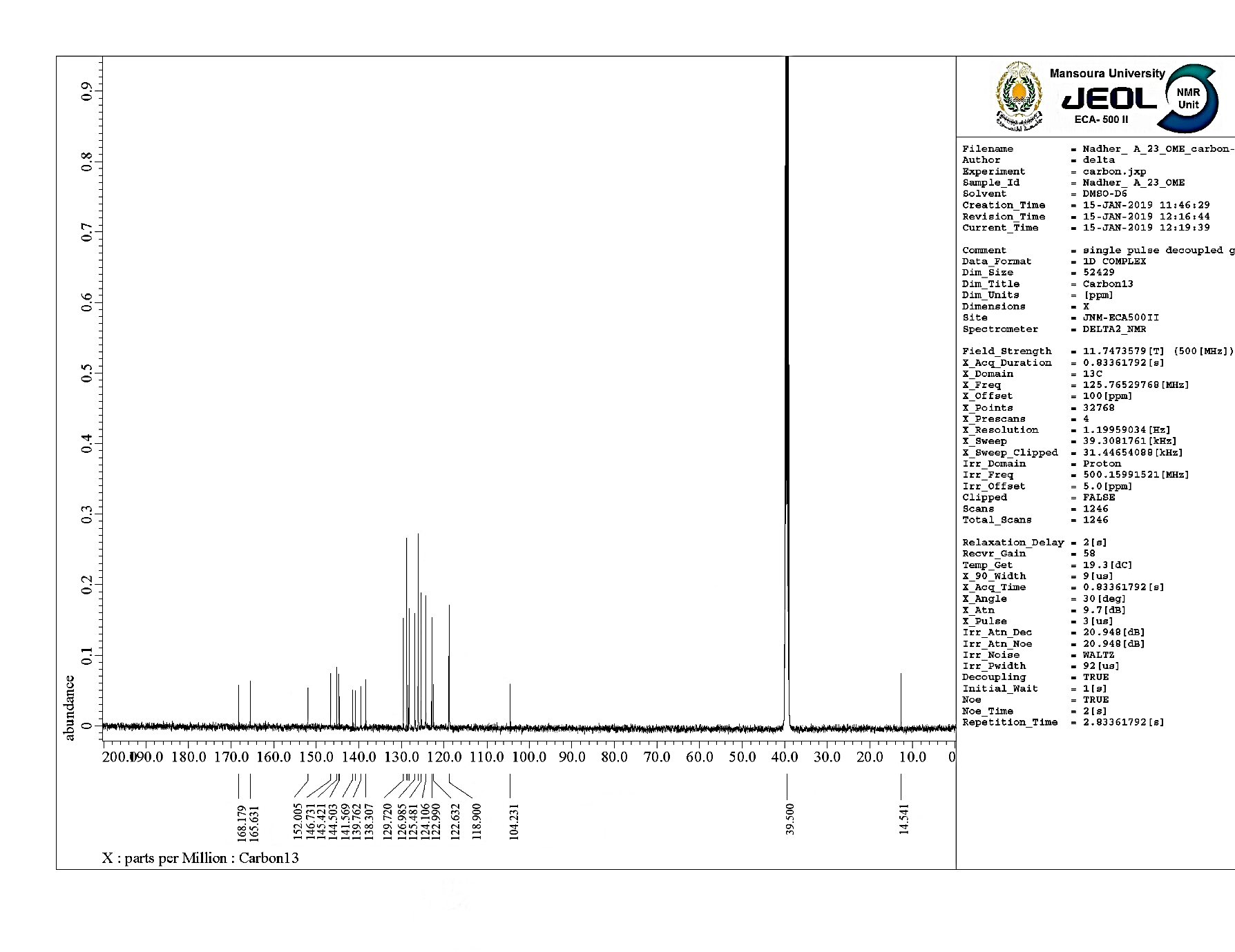
**

**Figure (S7): ^13^C NMR spectrum of compound 4a.**

**Figure (S8): Mas spectrum of compound 4a.**


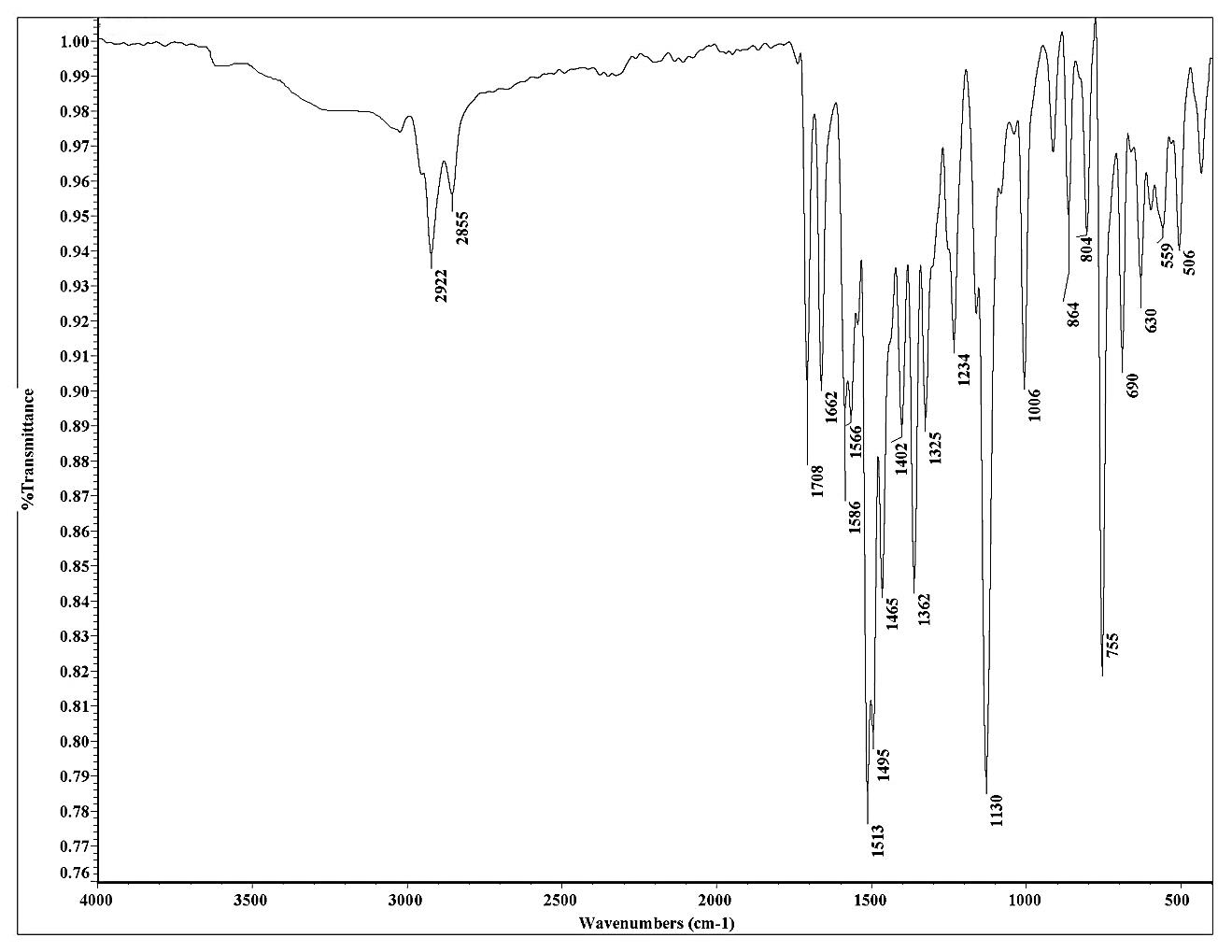

**Figure (S9): IR spectrum of compound 4b.**


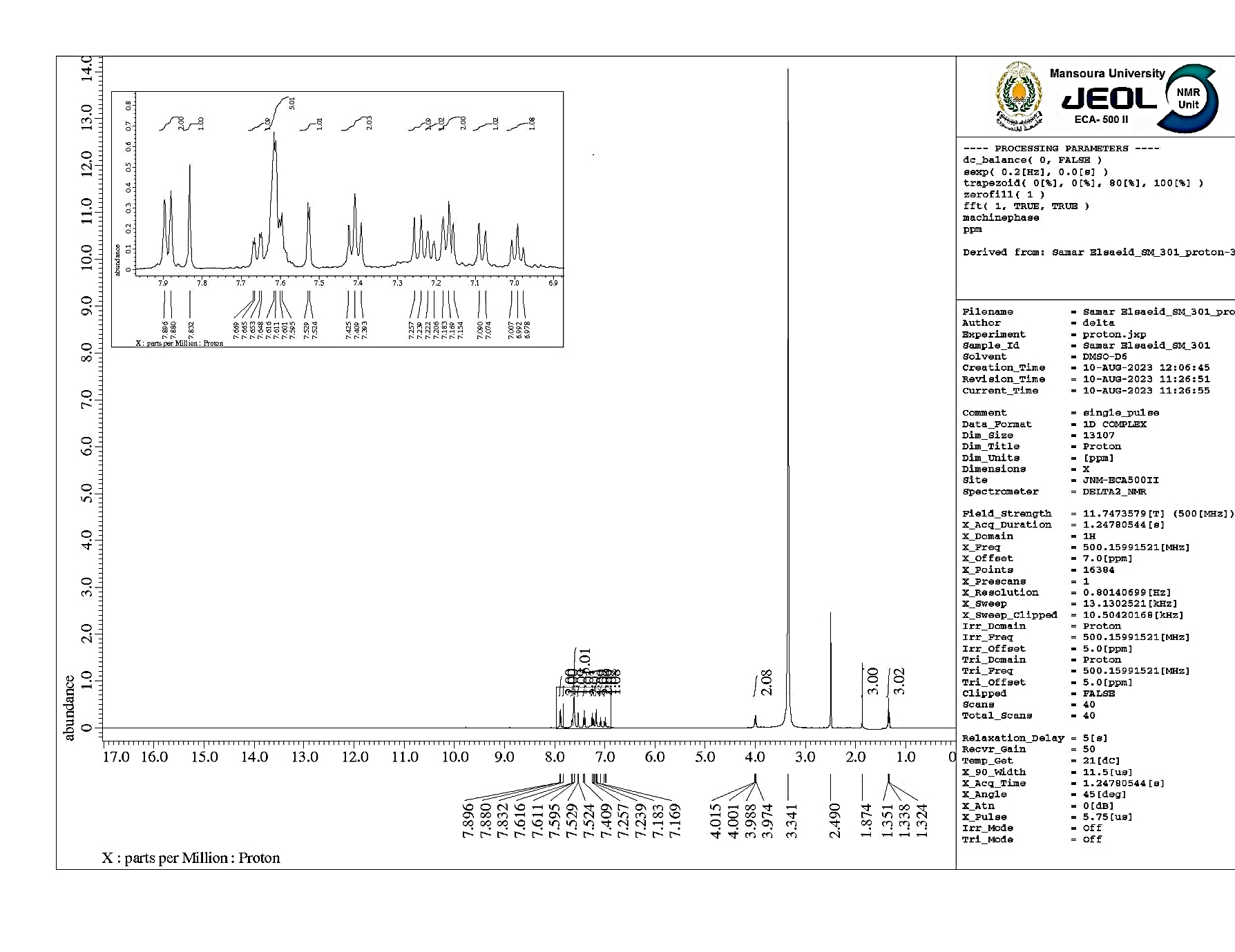

**Figure (S10): ^1^H NMR spectrum of compound 4b.**


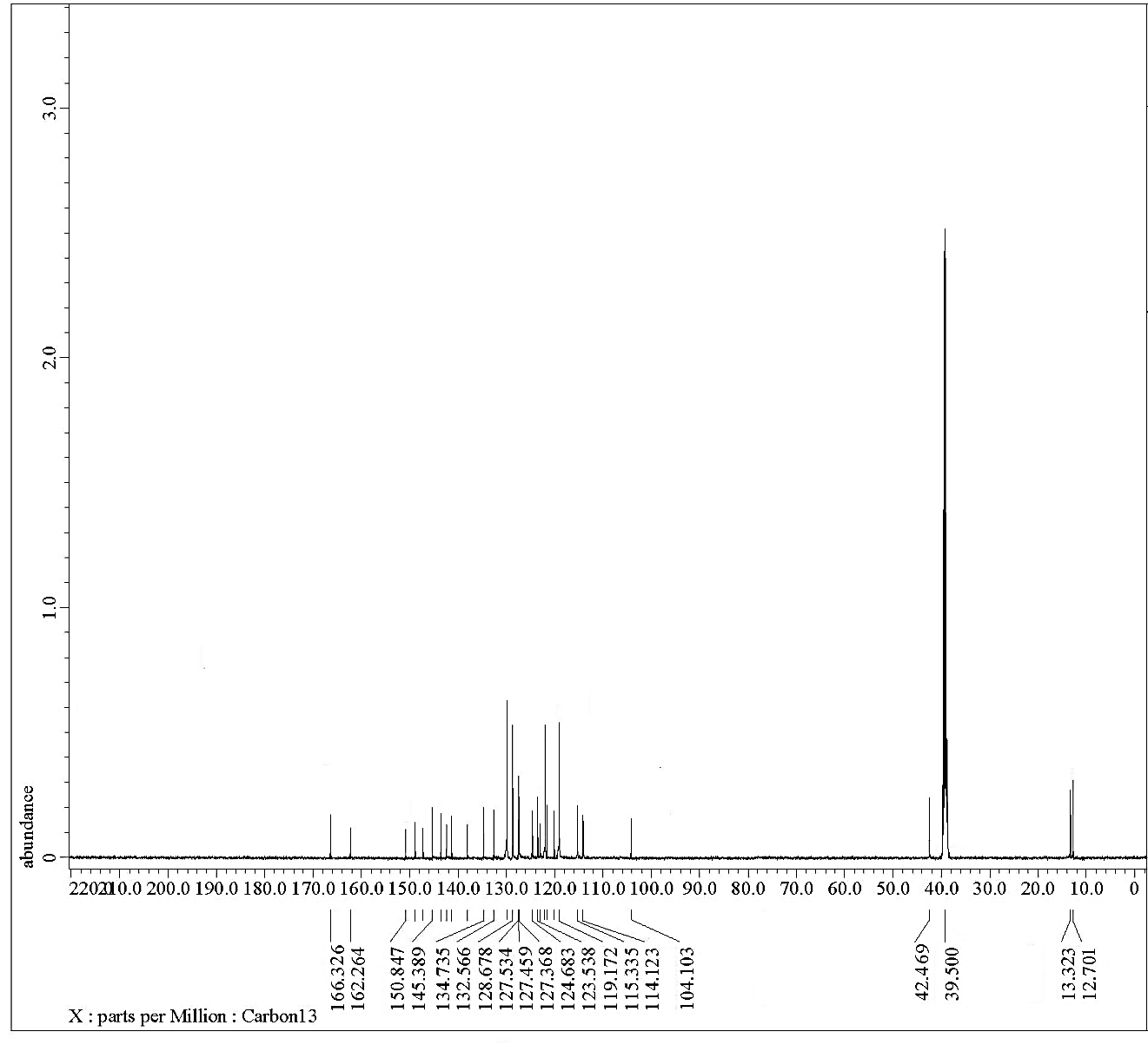

**Figure (S11): ^13^C NMR spectrum of compound 4b.**

**Figure (S12): Mass spectrum of compound 4b.**


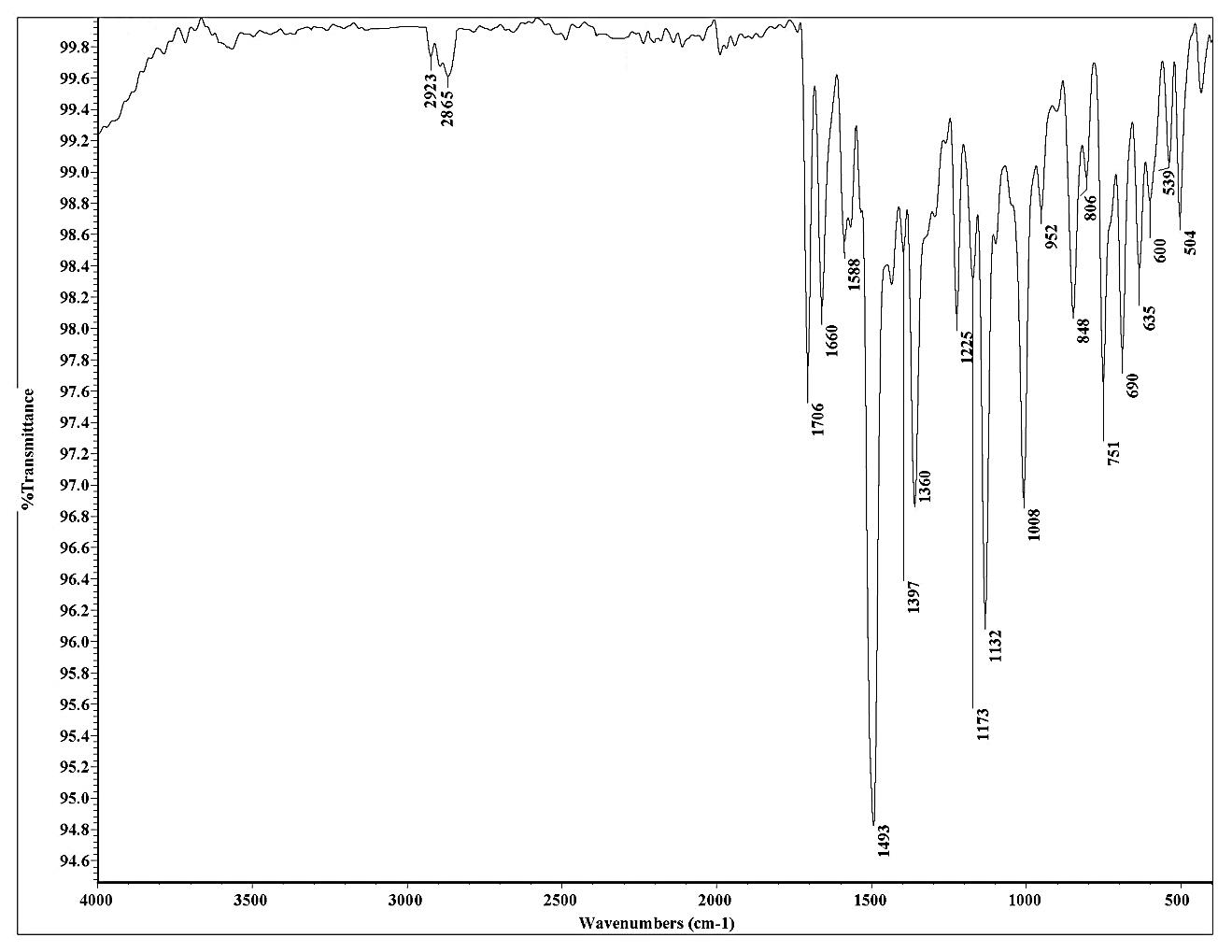

**Figure (S13): IR spectrum of compound 4c.**


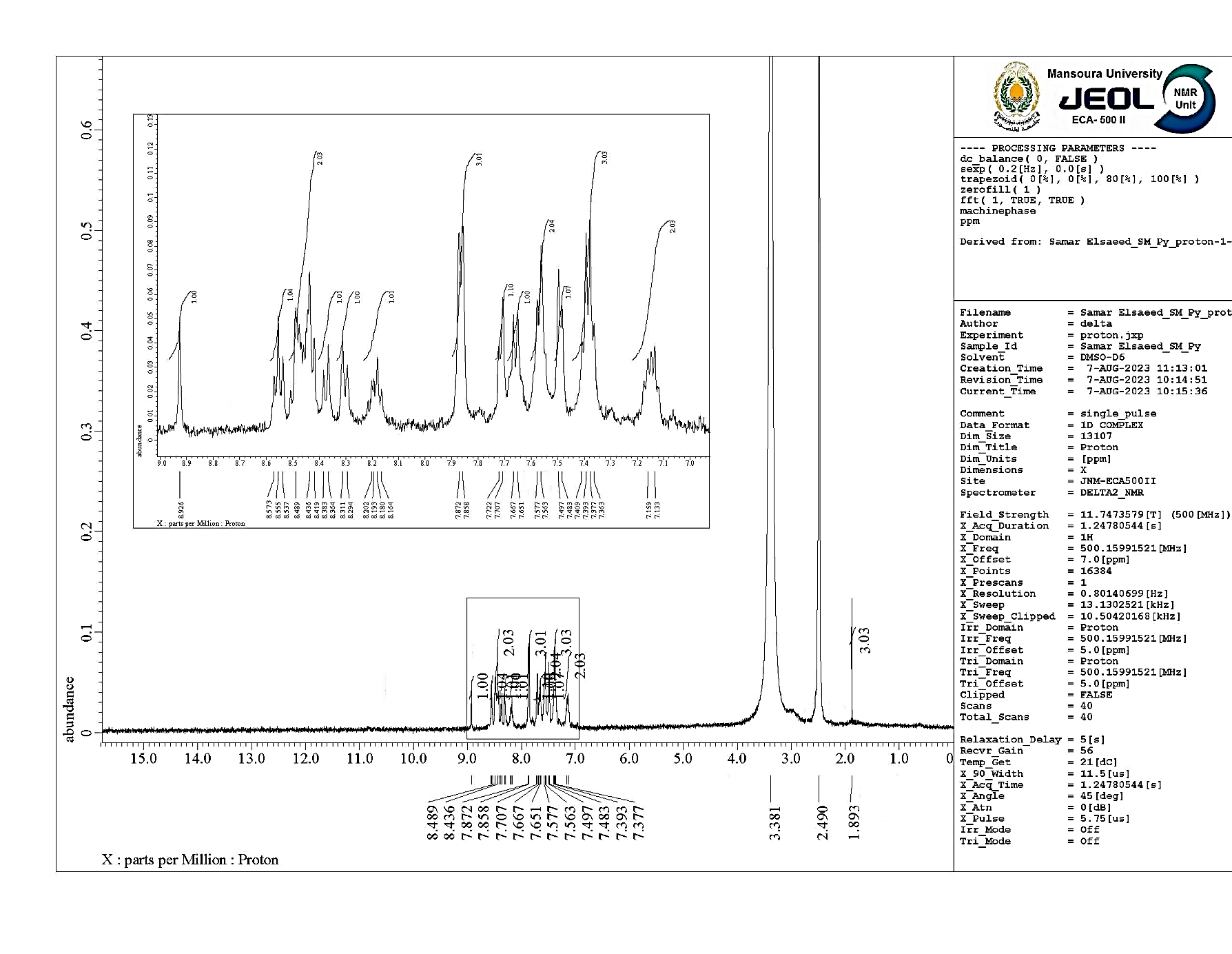

**Figure (S14): ^1^H NMR spectrum of compound 4c.**

**
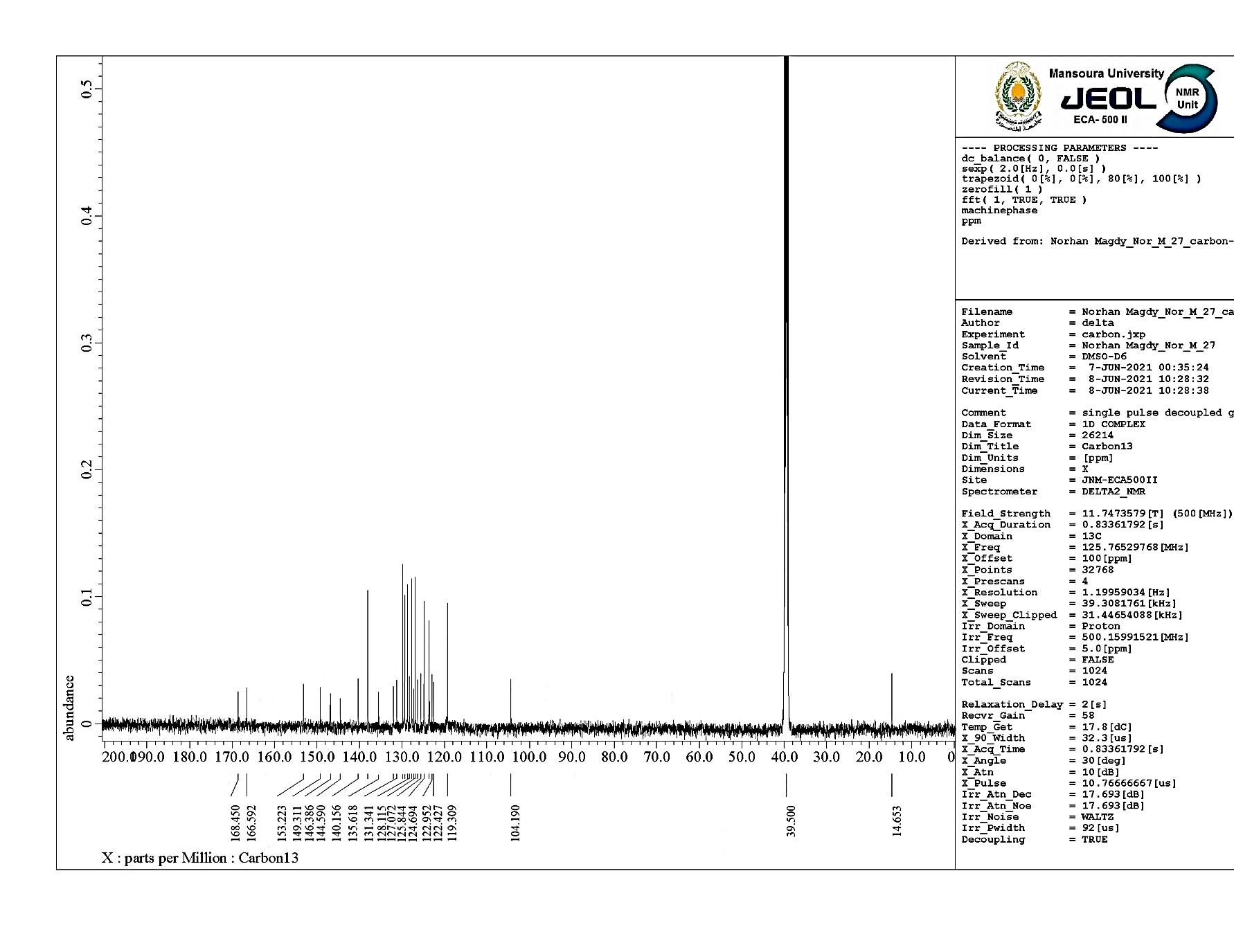
**

**Figure (S15): ^13^C NMR spectrum of compound 4c.**

**Figure (S16): Mass spectrum of compound 4c.**


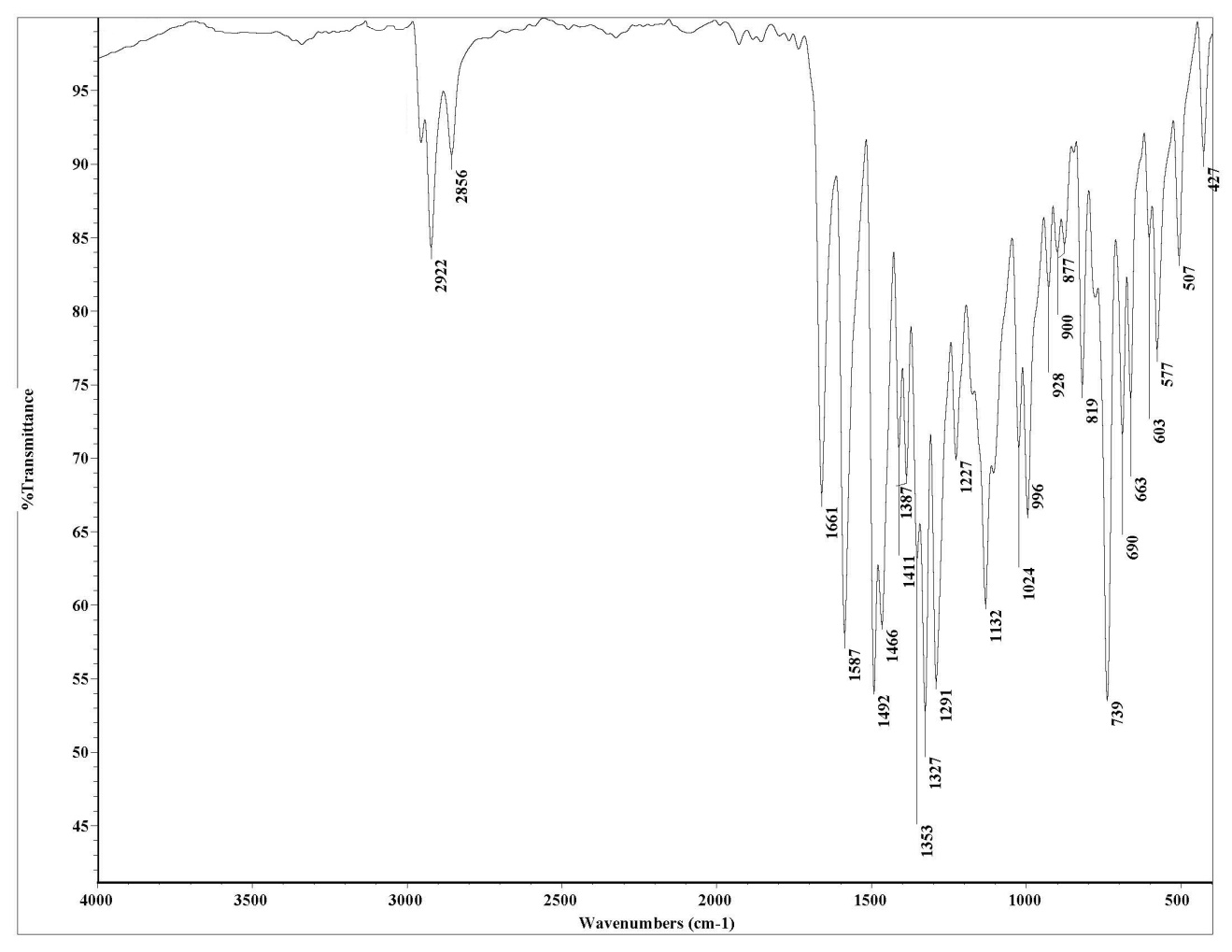

**Figure (S17): IR spectrum of compound 4d.**


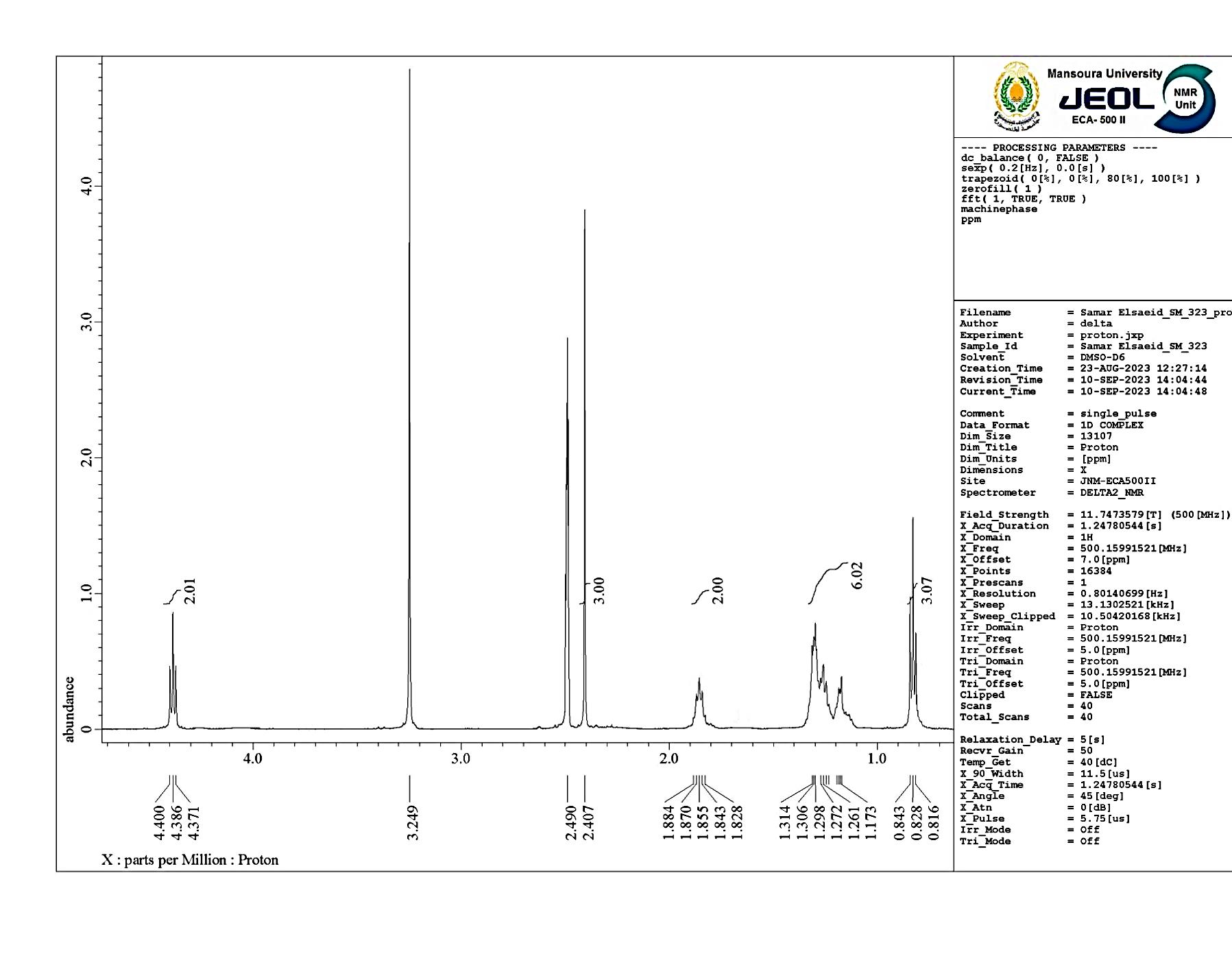

**Figure (S18): ^1^H NMR spectrum of compound 4d.**


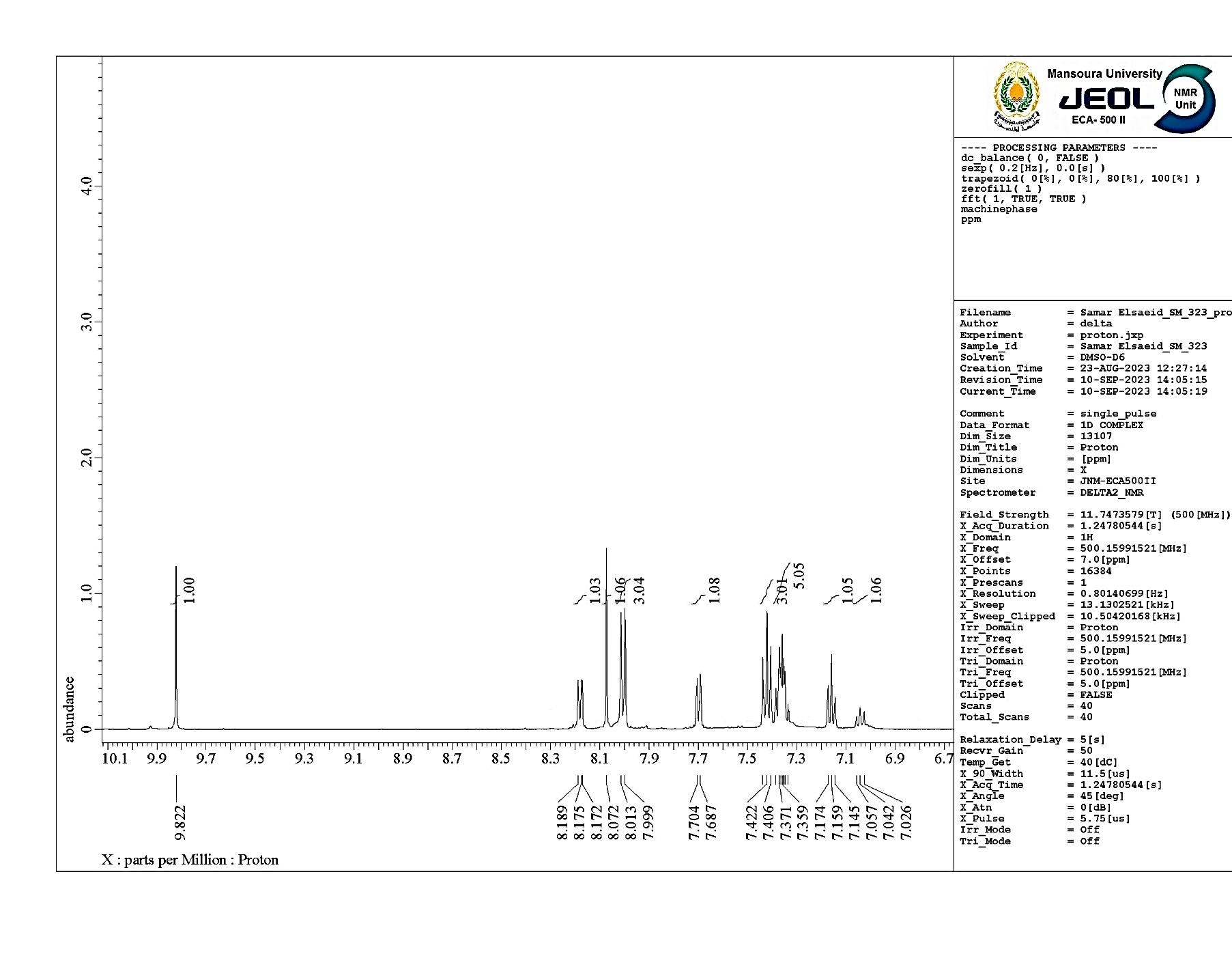

**Figure (S19): ^1^H NMR spectrum of compound 4d.**

**
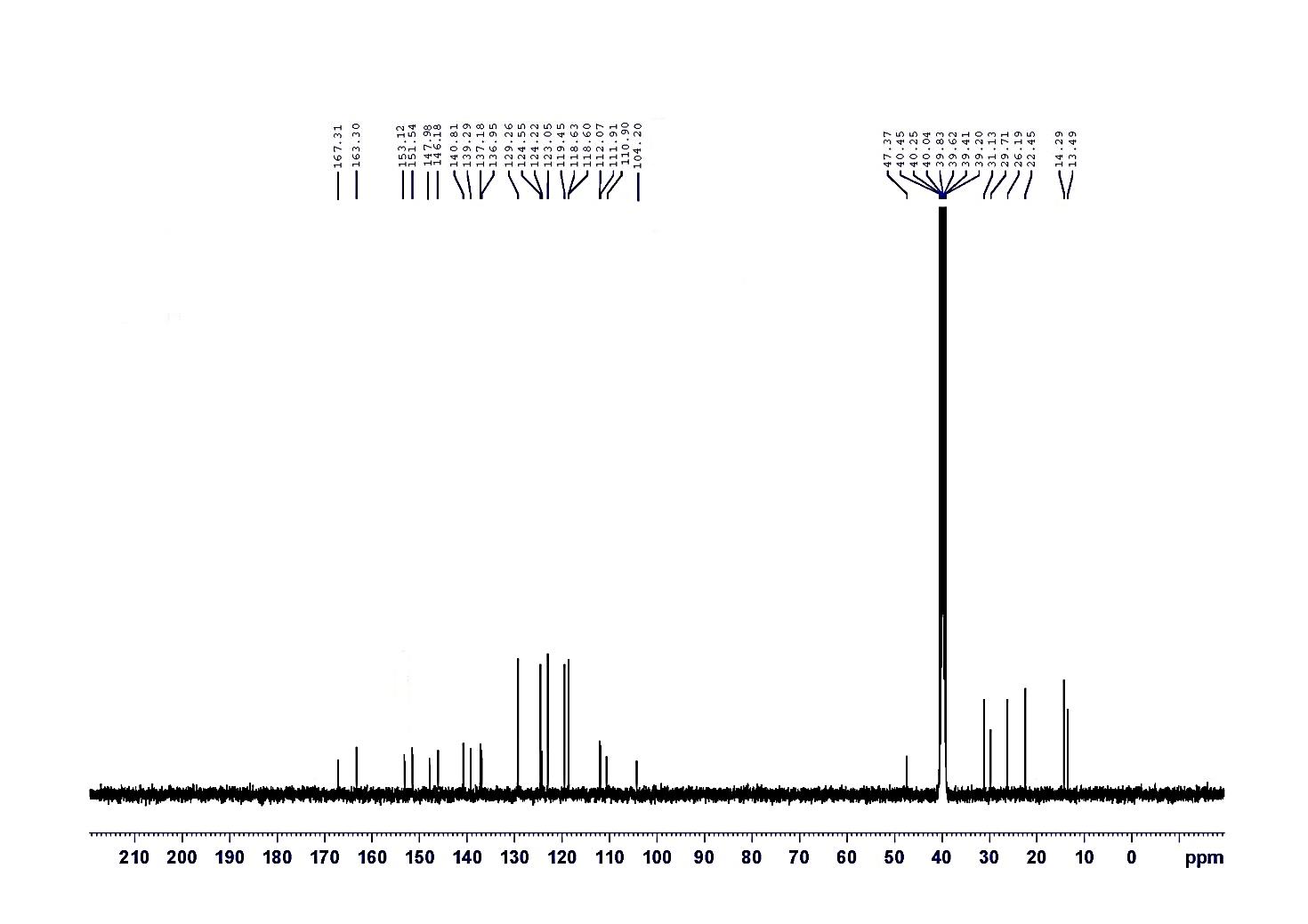
**

**Figure (S20): ^13^C NMR spectrum of compound 4d.**

**Figure (S21): Mass spectrum of compound 4d.**


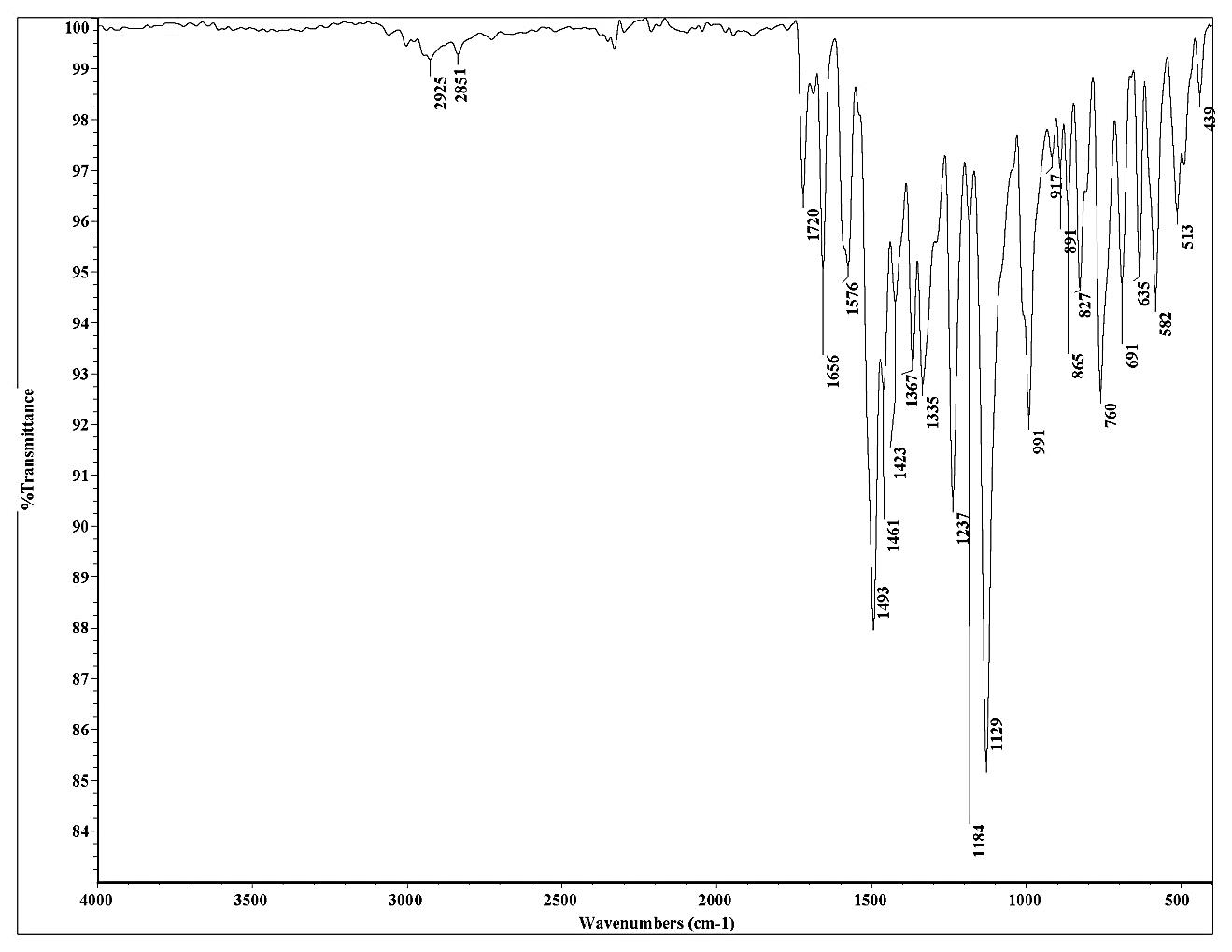

**Figure (S22): IR spectrum of compound 4e.**


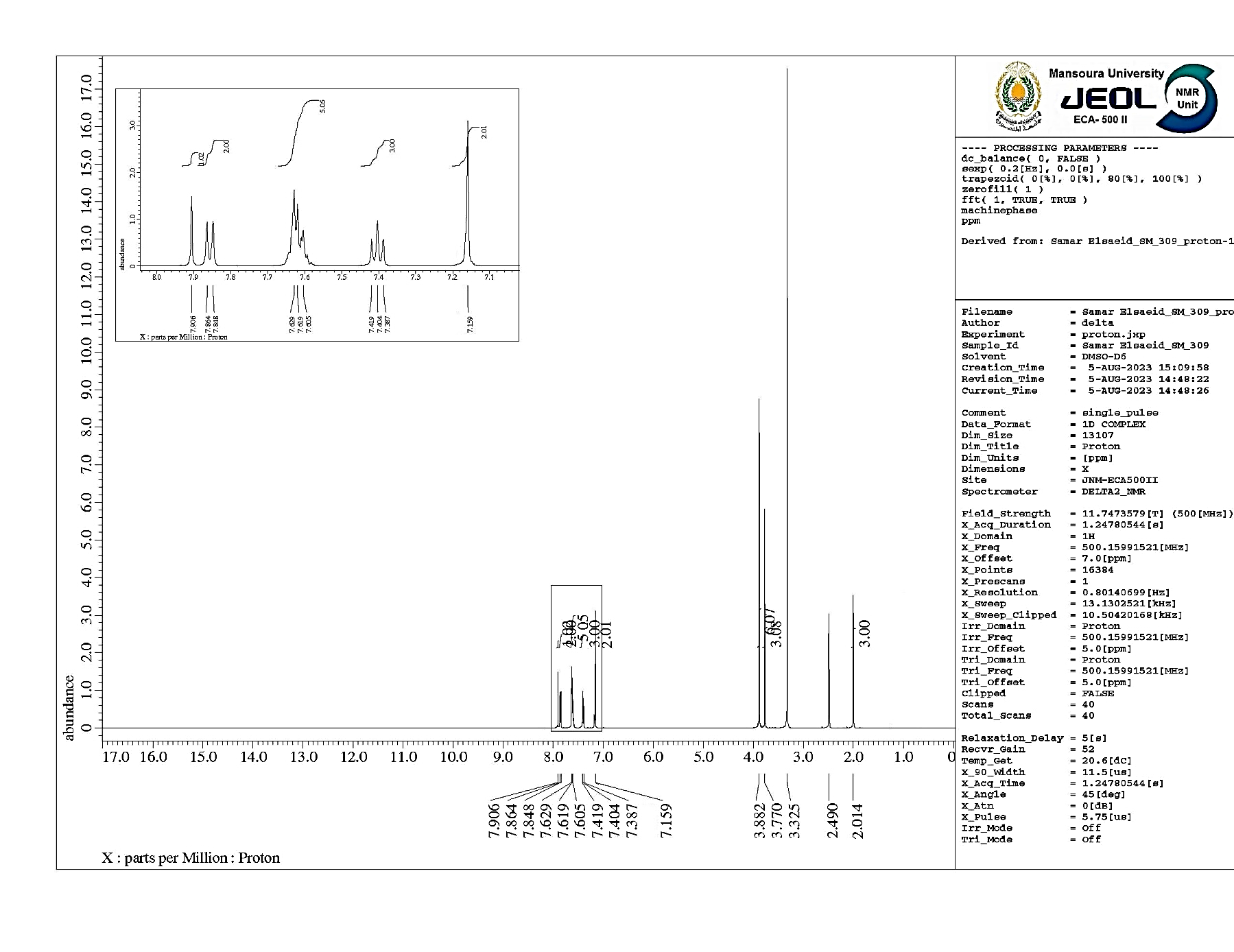

**Figure (S23): ^1^H NMR spectrum of compound 4e.**


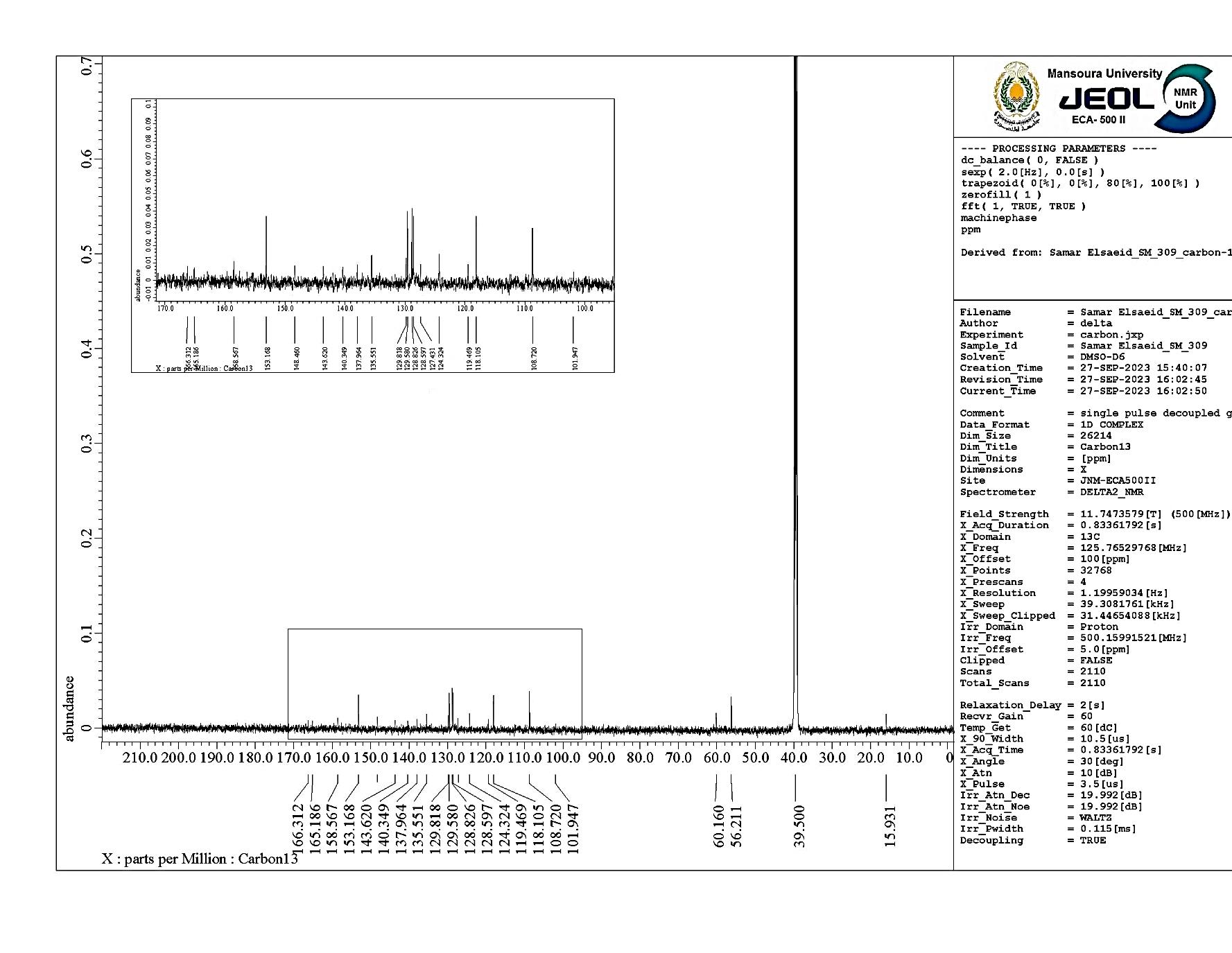

**Figure (S24): ^13^CNMR spectrum of compound 4e.**

**Figure (S25): Mass spectrum of compound 4e.**


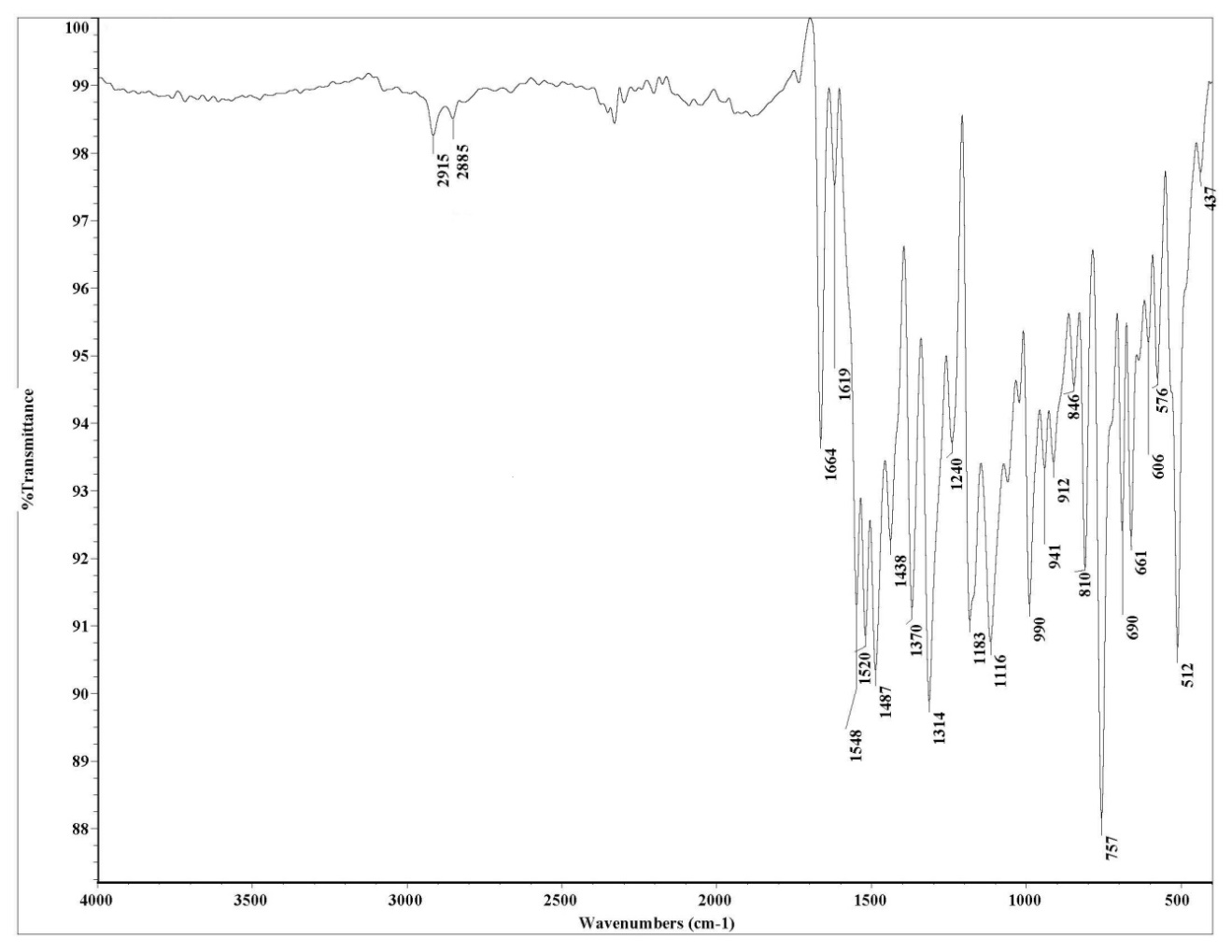

**Figure (S26): IR spectrum of compound 4f.**


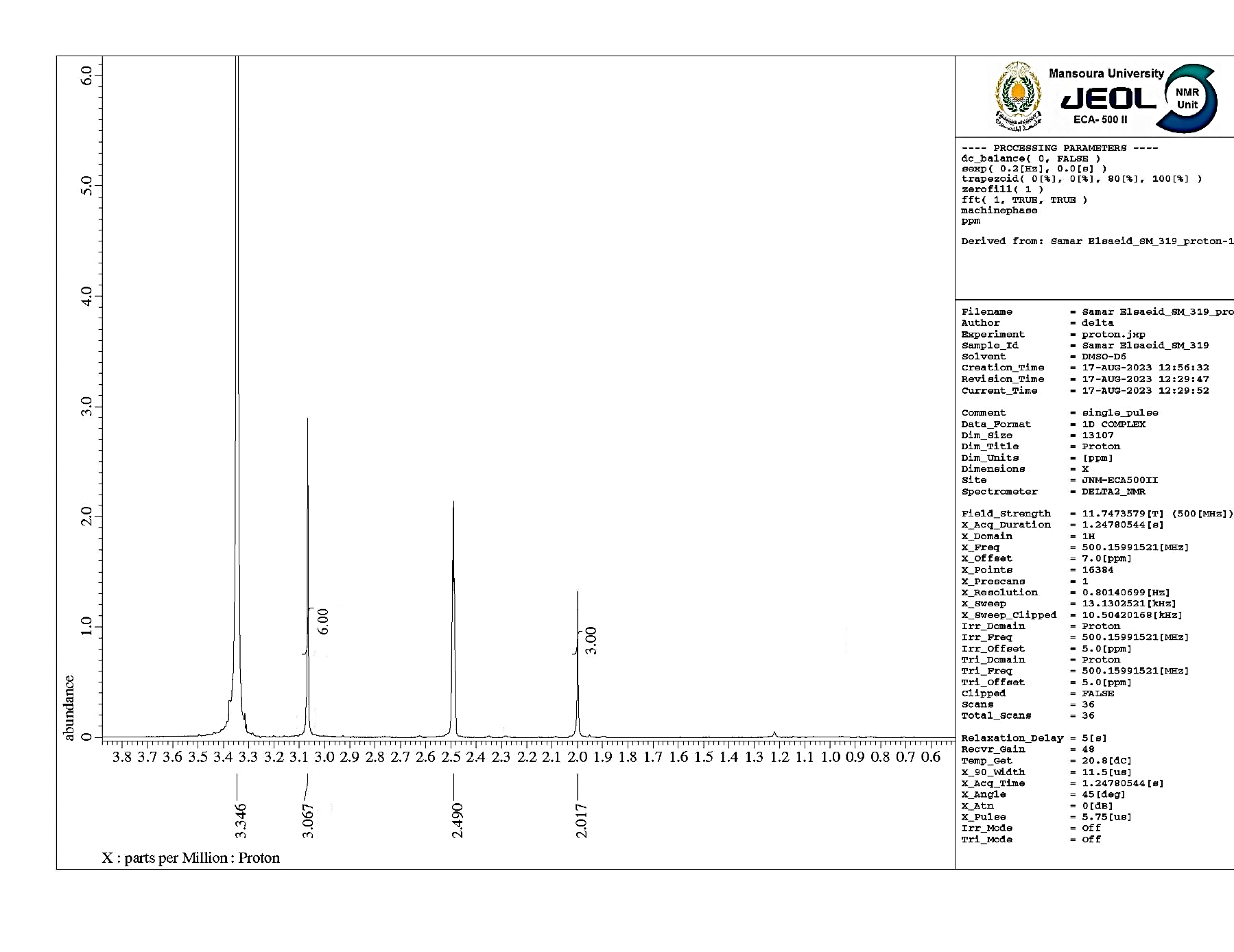

**Figure (S27): ^1^H NMR spectrum of compound 4f.**


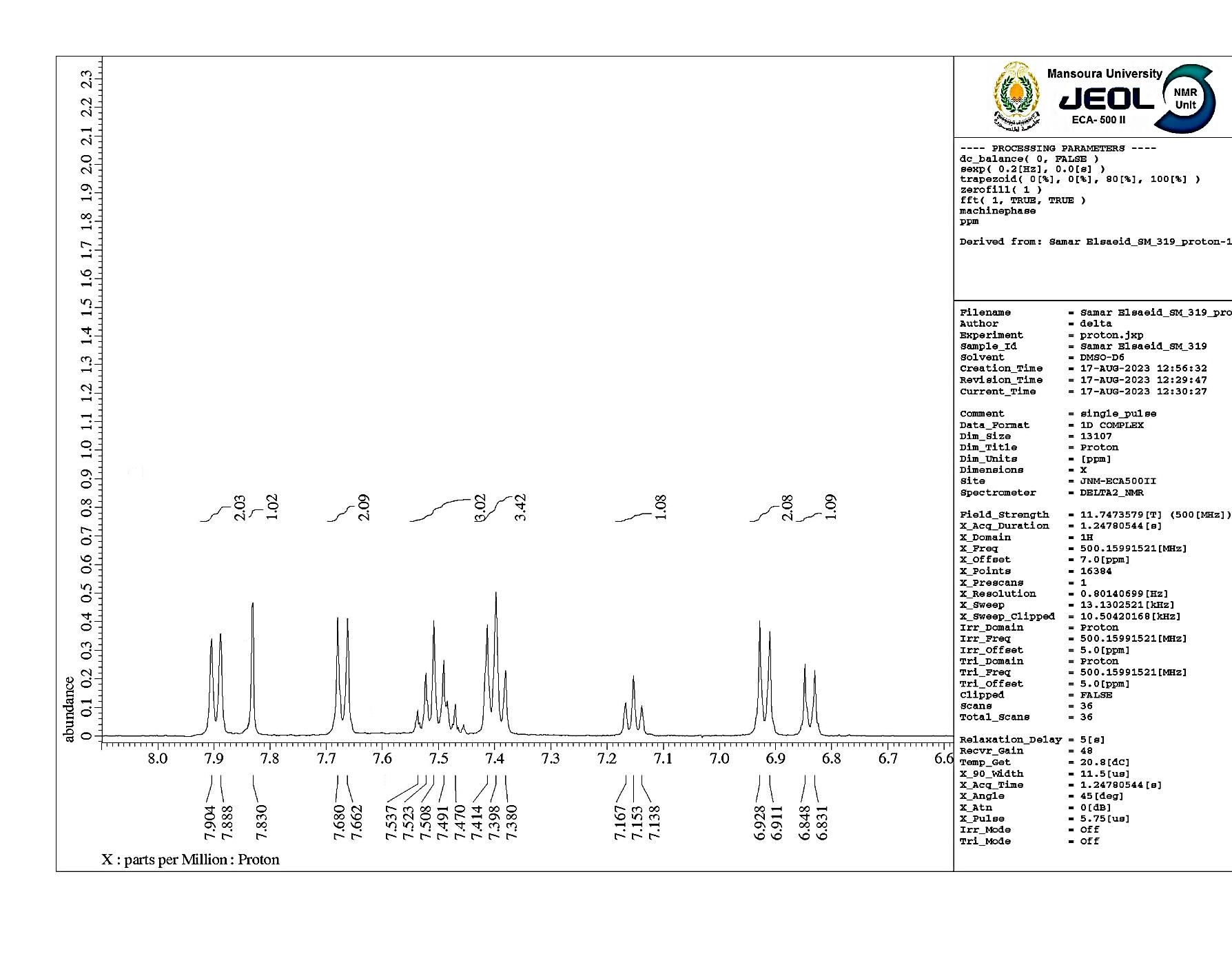

**Figure (S28): ^1^H NMR spectrum of compound 4f.**


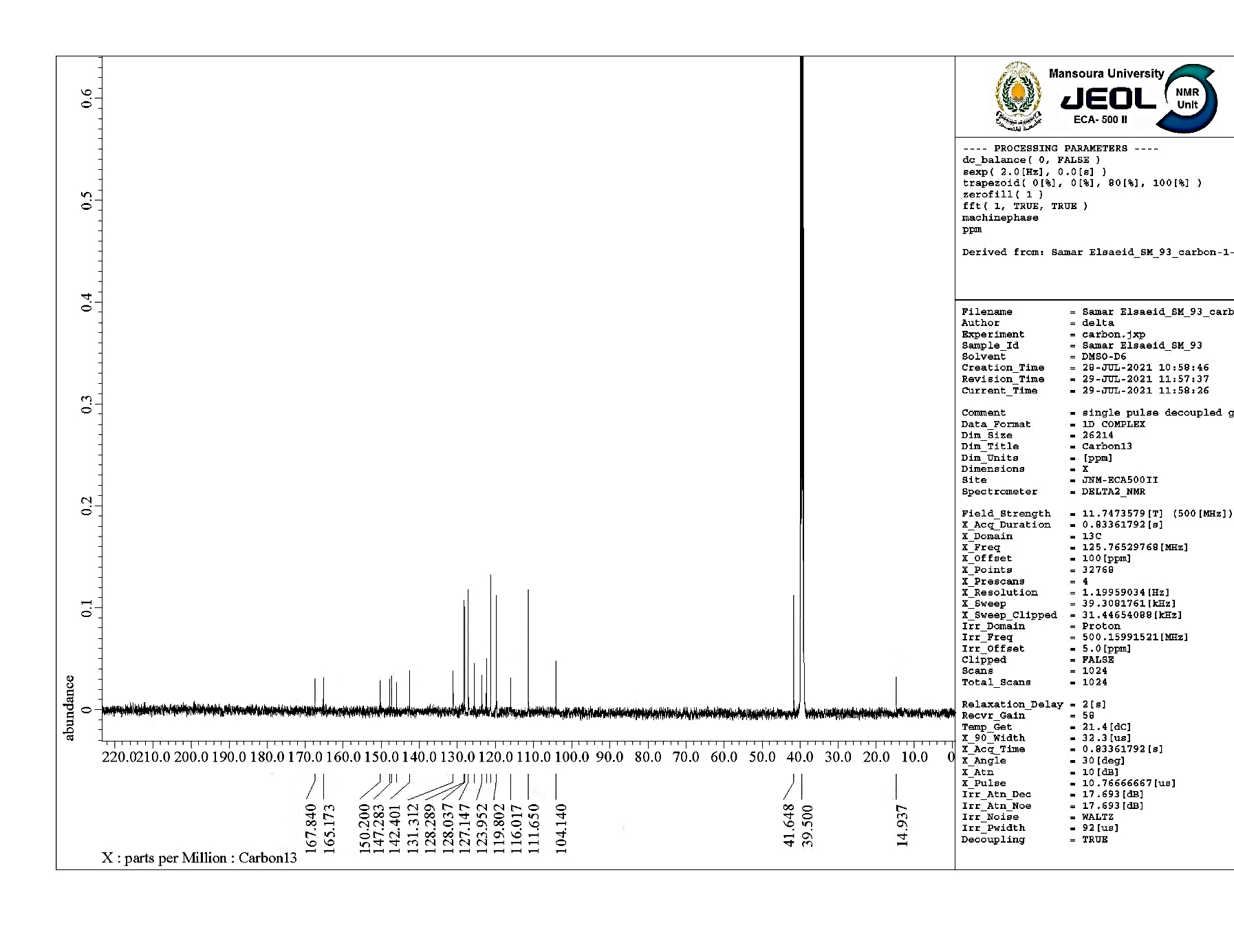

**Figure (S29): ^13^C NMR spectrum of compound 4f.**

**Figure (S30): Mass spectrum of compound 4f.**
